# Supplementary material for: Risk of all-cause mortality associated with chronic obstructive pulmonary disease and the role of healthy ageing trajectories: a population-based study of middle-aged and older adults
Source: BMJ Open. 2021 Jul 28;11(7):e050947. doi: 10.1136/bmjopen-2021-050947 (PMC8320253; doi:10.1136/bmjopen-2021-050947)

**Supplemental material**

**Supplementary Table 1.** Harmonised items of the healthy ageing scale in the SHARE study

| **ATHLOS harmonized variables** | | | **SHARE** |
|---|---|---|---|
| Memory | Self-reported ratings of memory at the time of the interview | Label | A4-B3 Memory self-rating |
| | | Values | 1 = None; 2 = Mild; 3 = Moderate; 4 = Severe; 5 = Extreme |
| | | Harmonisation | 1 = Absence; 2-5 = Presence |
| Immediate recall | Immediate recall of common nouns from a list | Label | ten words list learning first trial total |
| | | Values | Continuous Variable 0-10 |
| | | Harmonisation | <=25% into Presence<br>>25% into Absence |
| Delayed recall | Test that assesses delayed recall using the common nouns from the list previously employed for measuring Immediate recall | Label | ten words list learning delayed recall total |
| | | Values | Continuous Variable 0-10 |
| | | Harmonisation | <=25% into Presence<br>>25% into Absence |
| Verbal fluency† | Test that assesses verbal (semantic) fluency | Label | verbal fluency score |
| | | Values | Continuous Variable 0-88 |
| | | Harmonisation | <=25% into Presence<br>>25% into Absence |
| Orientation in time | Difficulties for orientation in time, evaluated by a set of questions about the date and day of the week | Label | orientation to date, month, year and day of week |
| | | Values | 0-3 = bad; 4 = good |
| | | Harmonisation | 4 = Absence; 0-3 = Presence |
| Numeracy | | Label | cf012_: Chance disease 10 perc. of 1000<br>cf013_: Half price [of a 300 Euro sofa]<br>cf014_: 6000 is two-thirds what is total price<br>cf015_: Amount in the savings account [on 2000 Euros after 2 years of 10% interest] |
| | | Values | All: 1 = correct answer: 100; 2 = wrong answer: 10; 3 = wrong answer: 90; 4 = wrong answer: 900; 97 = wrong answer: Other answer keep 1 into 1; recode -1, 2, 3, 4, 5, 6 and 97 into 0 |
| | | Harmonisation | All 1 = Absence; Some 0 = Presence |
| Sleeping | Sleeping problems | Label | sleep (part of EURO-D) |
| | | Values | 0 = Not selected; 1 = Selected |
| | | Harmonisation | 0 = Absence; 1 = Presence |
| Pain | It measures if the participant experiences some degree of pain or if the participant does not present any pain at all | Label | bothered by: pain in back, knees, hips or other joint |
| | | Values | 0 = Not selected; 1 = Selected |
| | | Harmonisation | 0 = Absence; 1 = Presence |
| Energy | Self-reported high level of energy experienced at the time of the interview | Label | fatigue |
| | | Values | 1 = Yes; 5 = No |
| | | Harmonisation | 5 = Absence; 1 = Presence |
| | | Label | bothered by: incontinence |

| | | | |
|---|---|---|---|
| Urine incontinence | It measures if the participant has experienced loss of urine (or has used any special device for urine leakage) | Values | 0 = Not selected; 1 = Selected |
| | | Harmonisation | 0 = Absence; 1 = Presence |
| Near vision | Difficulties for near vision | Label | eyesight reading |
| | | Values | 1 = excellent; 2 = very good; 3 = good; 4 = fair; 5 = poor |
| | | Harmonisation | 1-3 = Absence; 4-5 = Presence |
| Far vision | Difficulties for far vision | Label | eyesight distance |
| | | Values | 1 = excellent; 2 = very good; 3 = good; 4 = fair; 5 = poor |
| | | Harmonisation | 1-3 = Absence; 4-5 = Presence |
| Eyesight | Difficulties in eye sight using glasses or corrective lens as usual | Label | Is your eyesight (using glasses or contact lens if you use them) |
| | | Values | 1 = excellent; 2 = very good; 3 = good; 4 = fair; 5 = poor; 6 = registered or legally blind |
| | | Harmonisation | 1-3 = Absence; 4-6 = Presence |
| Hearing in general | It measures if the participant experiences some difficulty for hearing (i.e., hearing someone talking on the other side of the room in a normal voice) or not, using a hearing aid as usual | Label | hearing |
| | | Values | 1 = excellent; 2 = very good; 3 = good; 4 = fair; 5 = poor |
| | | Harmonisation | 1-3 = Absence; 4-5 = Presence |
| Hearing in a conversation | It measures if the participant experiences some difficulty (including total disability) for following a conversation (i.e., if there is a background noise, or several people talking) or not, using a hearing aid as usual | Label | hearing with one person |
| | | Values | 1 = Yes; 5 = No |
| | | Harmonisation | 1 = Absence; 0= Presence |
| Stooping, kneeling or crouching | Difficulty for stooping, kneeling or crouching | Label | difficulties: stooping, kneeling, crouching |
| | | Values | 0 = Not selected; 1 = Selected |
| | | Harmonisation | 0 = Absence; 1 = Presence |
| Lifting or carrying weights | Difficulty for lifting or carrying weights | Label | difficulties: lifting or carrying weights over 5 kilos |
| | | Values | 0 = Not selected; 1 = Selected |
| | | Harmonisation | 0 = Absence; 1 = Presence |
| Climbing stairs | Difficulty for climbing stairs | Label | difficulties: climbing one flight of stairs |
| | | Values | 0 = Not selected; 1 = Selected |
| | | Harmonisation | 0 = Absence; 1 = Presence |
| Getting up | Difficulty for getting up from sitting down | Label | difficulties: getting up from chair |
| | | Values | 0 = Not selected; 1 = Selected |
| | | Harmonisation | 0 = Absence; 1 = Presence |
| Walking | Difficulty for walking by yourself and without using any special equipment | Label | difficulties: walking 100 metres |
| | | Values | 0 = Not selected; 1 = Selected |
| | | Harmonisation | 0 = Absence; 1 = Presence |
| Pulling or pushing | Difficulty for pulling or pushing large objects | Label | difficulties: pulling or pushing large objects |
| | | Values | 0 = Not selected; 1 = Selected |
| | | Harmonisation | 0 = Absence; 1 = Presence |
| Sitting | Difficulty for sitting for long periods | Label | difficulties: sitting two hours |
| | | Values | 0 = Not selected; 1 = Selected |

| | | | |
|---|---|---|---|
| | | Harmonisation | 0 = Absence; 1 = Presence |
| Reaching or extending arms | Difficulty for reaching / extending arms | Label | difficulties: reaching or extending arms above shoulder |
| | | Values | 0 = Not selected; 1 = Selected |
| | | Harmonisation | 0 = Absence; 1 = Presence |
| Walking speed | It is measured assessing the time that is taken to walk a distance | Label | walking speed |
| | | Values | Continuous |
| | | Harmonisation | <=25% into Presence<br>>25% into Absence |
| Dizziness | Dizziness problems when walking on a level surface | Label | bothered by: dizziness, faints or blackouts |
| | | Values | 0 = Not selected; 1 = Selected |
| | | Harmonisation | 0 = Absence; 1 = Presence |
| Picking up | Difficulty for picking up things with fingers, e.g. picking up a coin | Label | difficulties: picking up a small coin from a table |
| | | Values | 0 = Not selected; 1 = Selected |
| | | Harmonisation | 0 = Absence; 1 = Presence |
| Getting in or out of bed | Difficulty for getting in or out of bed | Label | difficulties: getting in or out of bed |
| | | Values | 0 = Not selected; 1 = Selected |
| | | Harmonisation | 0 = Absence; 1 = Presence |
| Bathing or showering | Difficulties for bathing or showering | Label | difficulties: bathing or showering |
| | | Values | 0 = Not selected; 1 = Selected |
| | | Harmonisation | 0 = Absence; 1 = Presence |
| Getting dressed | Difficulty for getting dressed | Label | difficulties: dressing, including shoes and socks |
| | | Values | 0 = Not selected; 1 = Selected |
| | | Harmonisation | 0 = Absence; 1 = Presence |
| Moving around the home | Difficulty for moving around the home | Label | difficulties: walking across a room |
| | | Values | 0 = Not selected; 1 = Selected |
| | | Harmonisation | 0 = Absence; 1 = Presence |
| Toilet | Difficulties for using the toilet | Label | difficulties: using the toilet, incl getting up or down |
| | | Values | 0 = Not selected; 1 = Selected |
| | | Harmonisation | 0 = Absence; 1 = Presence |
| Eating | Difficulties for eating | Label | difficulties: eating, cutting up food |
| | | Values | 0 = Not selected; 1 = Selected |
| | | Harmonisation | 0 = Absence; 1 = Presence |
| Housework | Difficulties for doing housework | Label | difficulties: doing work around the house or garden |
| | | Values | 0 = Not selected; 1 = Selected |
| | | Harmonisation | 0 = Absence; 1 = Presence |
| Shopping | Difficulties for shopping groceries | Label | difficulties: shopping for groceries |
| | | Values | 0 = Not selected; 1 = Selected |
| | | Harmonisation | 0 = Absence; 1 = Presence |
| Meals | Difficulties in preparing meals | Label | difficulties: preparing a hot meal |
| | | Values | 0 = Not selected; 1 = Selected |
| | | Harmonisation | 0 = Absence; 1 = Presence |
| Map | Difficulties for using a map | Label | difficulties: using a map in a strange place |
| | | Values | 0 = Not selected; 1 = Selected |
| | | Harmonisation | 0 = Absence; 1 = Presence |
| Money | Difficulties for managing money, bills, or expenses | Label | difficulties: managing money |
| | | Values | 0 = Not selected; 1 = Selected |
| | | Harmonisation | 0 = Absence; 1 = Presence |
| Medications | Difficulties for taking medications | Label | difficulties: taking medications |

| | | | |
|---|---|---|---|
| | | Values | 0 = Not selected; 1 = Selected |
| | | Harmonisation | 0 = Absence; 1 = Presence |
| Telephone | Difficulties for using telephone | Label | difficulties: telephone calls |
| | | Values | 0 = Not selected; 1 = Selected |
| | | Harmonisation | 0 = Absence; 1 = Presence |

**Supplementary Table 2.** Results from the latent class growth analysis models for the three birth cohorts

| No. of classes | Loglik | npm | BIC | % class 1 | % class 2 | % class 3 | % class 4 | % class 5 |
|---|---|---|---|---|---|---|---|---|
| | | | | **≤1935** | | | | |
| 1 | -83923.67 | 3 | 167875.0 | 100.00 | | | | |
| 2 | -80924.65 | 6 | 161904.6 | 51.39 | 48.60 | | | |
| **3** | **-79920.76** | **9** | **159924.4** | **20.21** | **31.38** | **48.39** | | |
| 4 | -79545.29 | 12 | 159201.1 | 7.37 | 32.59 | 23.22 | 36.80 | |
| 5 | -79429.36 | 15 | 158996.8 | 31.64 | 23.08 | 1.31 | 7.19 | 36.76 |
| | | | | **1936 – 1945** | | | | |
| **No. of classes** | **Loglik** | **npm** | **BIC** | **% class 1** | **% class 2** | **% class 3** | **% class 4** | **% class 5** |
| 1 | -86218.80 | 3 | 172465.1 | 100.00 | | | | |
| 2 | -82811.31 | 6 | 165677.6 | 66.84 | 33.15 | | | |
| **3** | **-81877.93** | **9** | **163838.3** | **49.72** | **12.44** | **37.83** | | |
| 4 | -81611.24 | 12 | 163332.3 | 43.55 | 35.79 | 2.38 | 18.26 | |
| 5 | -81540.03 | 15 | 163217.4 | 33.21 | 12.13 | 24.67 | 28.00 | 1.97 |
| | | | | **>1945** | | | | |
| **No. of classes** | **Loglik** | **npm** | **BIC** | **% class 1** | **% class 2** | **% class 3** | **% class 4** | **% class 5** |
| 1 | -89325.74 | 3 | 178679.1 | 100.00 | | | | |
| 2 | -85721.95 | 6 | 171499.2 | 23.37 | 76.62 | | | |
| **3** | **-84856.16** | **9** | **169795.3** | **8.75** | **30.81** | **60.43** | | |
| 4 | -84608.23 | 12 | 169327.1 | 30.97 | 53.19 | 13.34 | 2.47 | |
| 5 | -84607.84 | 15 | 169354.0 | 2.39 | 13.17 | 32.36 | 52.02 | 0.02 |

**Supplementary Figure 1.** Trajectories of healthy ageing among birth cohorts

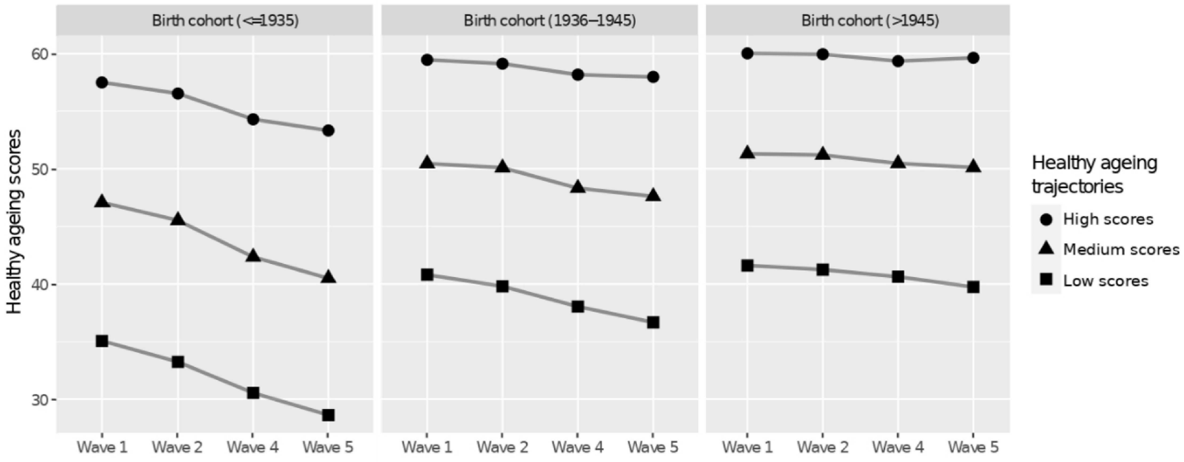

**Supplementary Figure 2.** Estimates of cumulative excess of risk of COPD and healthy ageing trajectories, separately by using the Aalen's additive regression

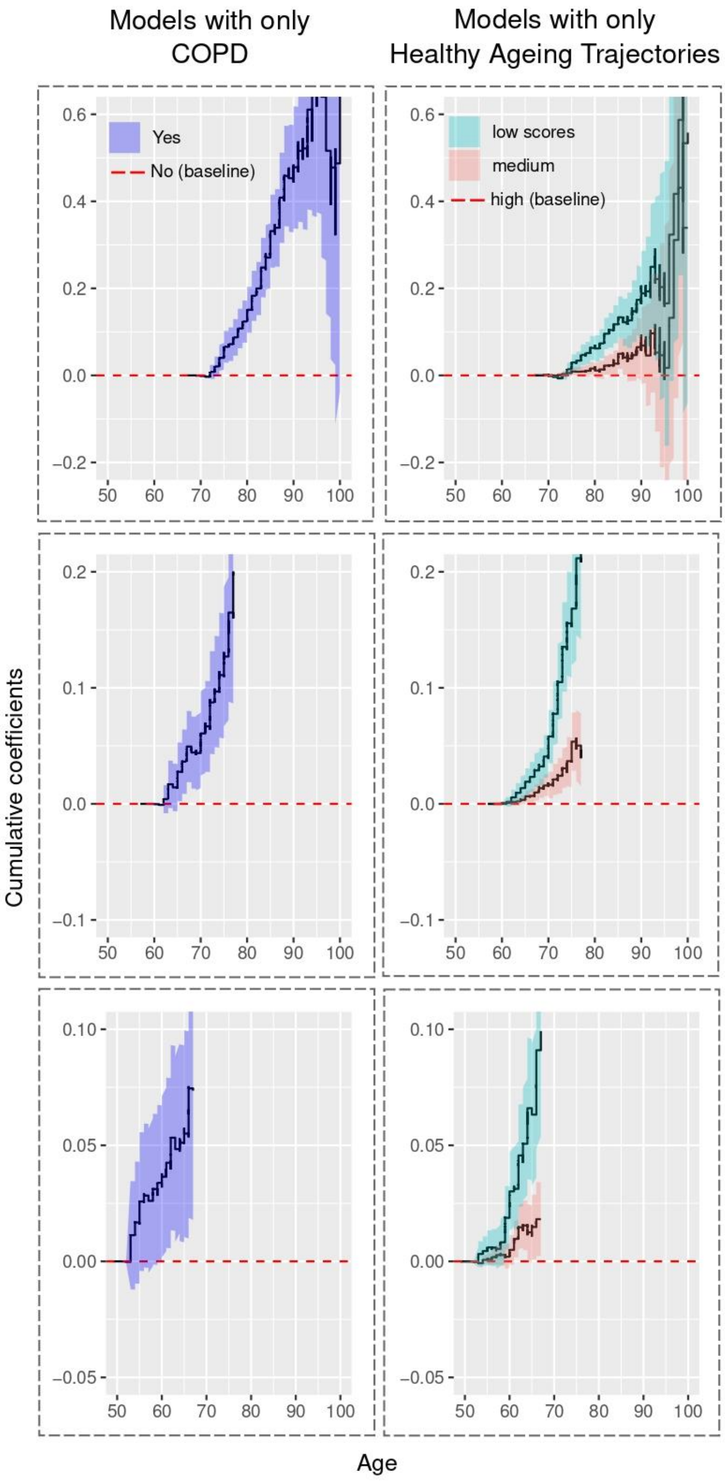




Bayes-Marin I, *et al. BMJ Open* 2021; 11:e050947. doi: 10.1136/bmjopen-2021-050947

**Supplementary Figure 3.**

**Figure 3.1.** Estimates of cumulative excess risk of covariates from the Aalen's additive regression model by >1945 birth cohort sub-sample.

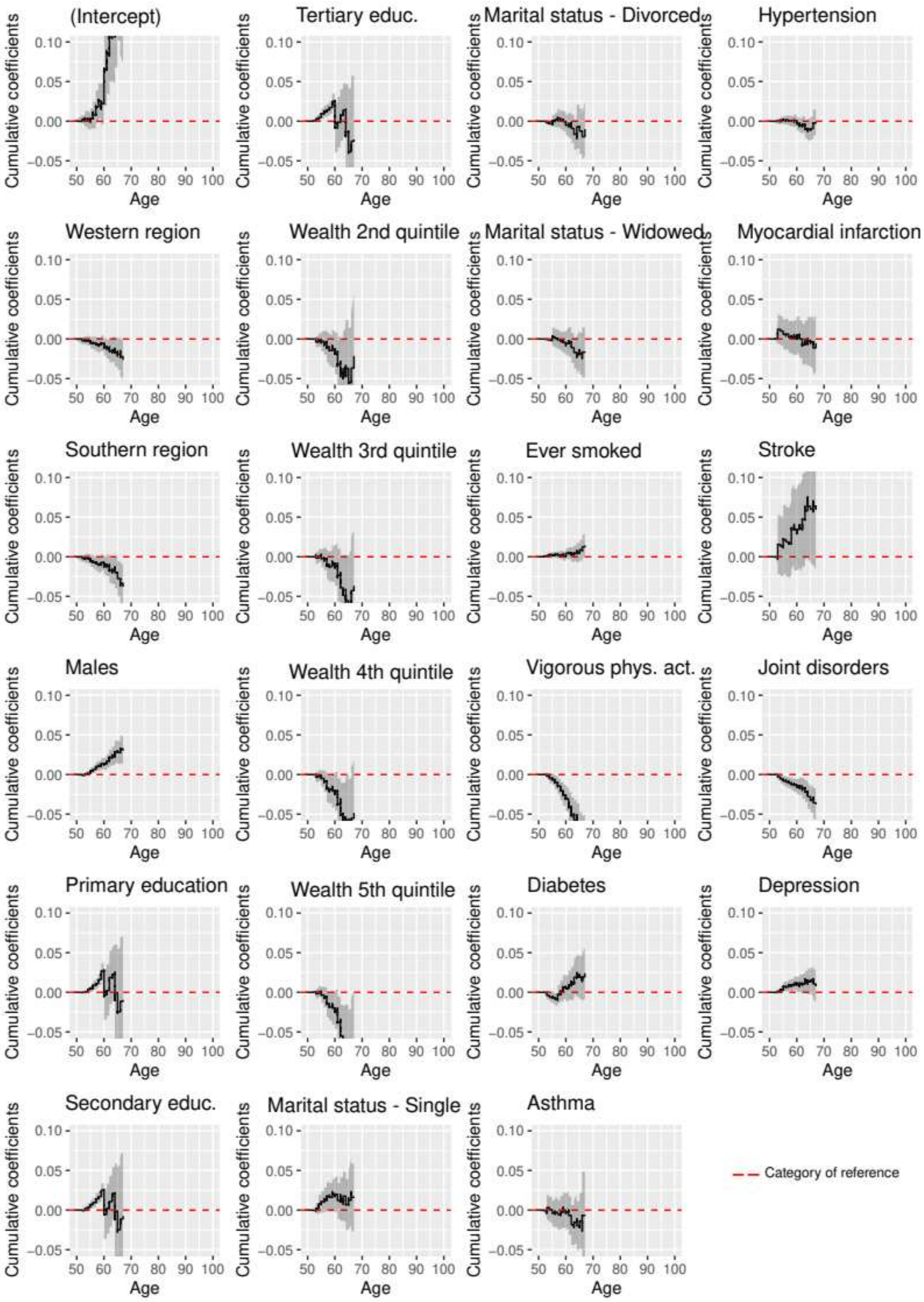

**Figure 3.2.** Estimates of cumulative excess risk of covariates from the Aalen's additive regression model by 1936-1945 birth cohort sub-sample.

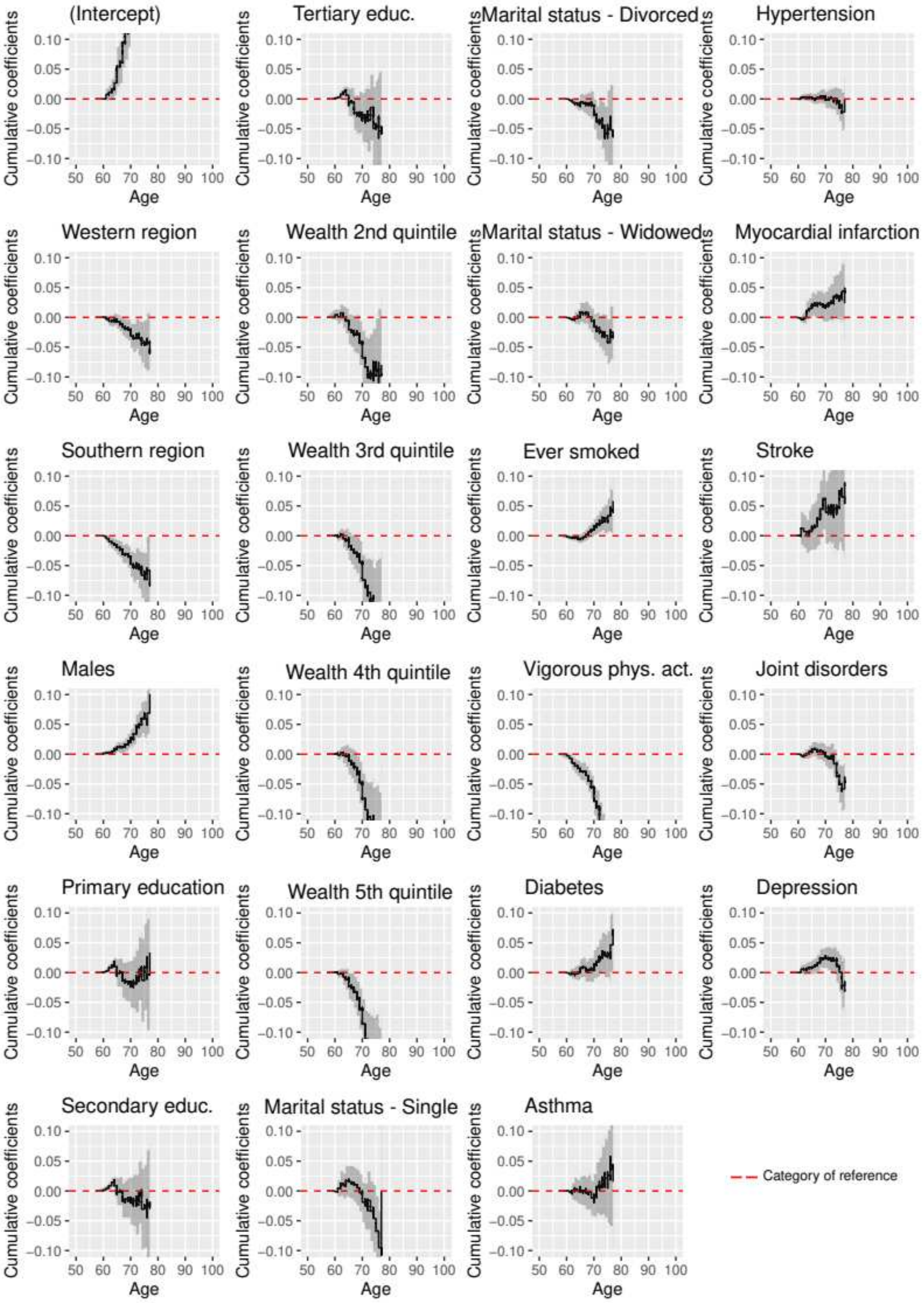

**Figure 3.3.** Estimates of cumulative excess risk of covariates from the Aalen's additive regression model by ≤1935 birth cohort sub-sample.

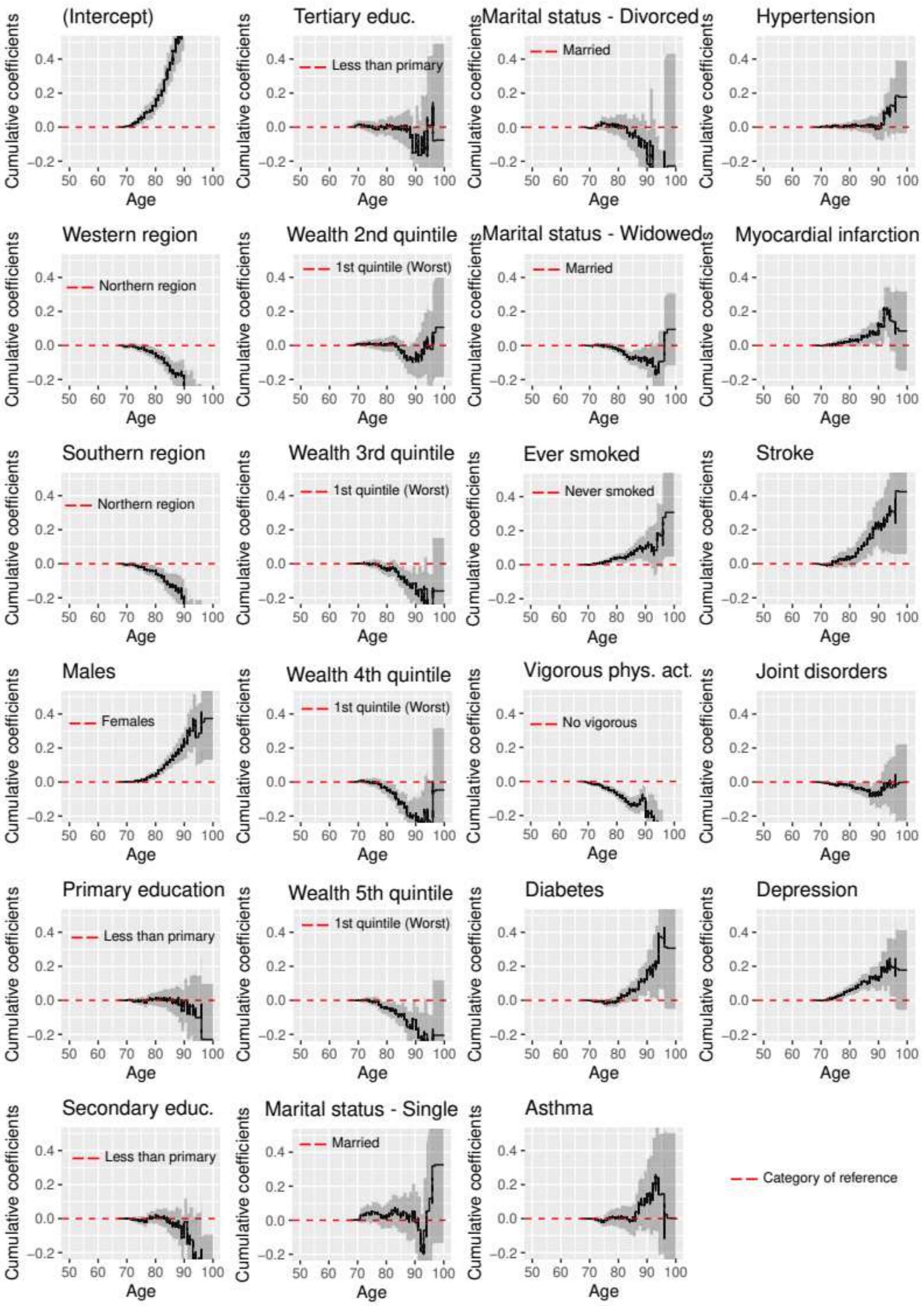

Supplement: Supplementary data [file bmjopen-2021-050947supp001.pdf]
