# Supplementary material for: Risk of all-cause mortality associated with chronic obstructive pulmonary disease and the role of healthy ageing trajectories: a population-based study of middle-aged and older adults
Source: BMJ Open. 2021 Jul 28;11(7):e050947. doi: 10.1136/bmjopen-2021-050947 (PMC8320253; doi:10.1136/bmjopen-2021-050947)
Supplement: Author's manuscript [file bmjopen-2021-050947.draft_revisions.pdf]

BMJ Open is committed to open peer review. As part of this commitment we make the peer review history of every article we publish publicly available.

When an article is published we post the peer reviewers' comments and the authors' responses online. We also post the versions of the paper that were used during peer review. These are the versions that the peer review comments apply to.

The versions of the paper that follow are the versions that were submitted during the peer review process. They are not the versions of record or the final published versions. They should not be cited or distributed as the published version of this manuscript.

BMJ Open is an open access journal and the full, final, typeset and author-corrected version of record of the manuscript is available on our site with no access controls, subscription charges or pay-per-view fees (http://bmjopen.bmj.com).

If you have any questions on BMJ Open's open peer review process please email
info.bmjopen@bmj.com

BMJ Open

Risk of all-cause mortality associated with chronic obstructive pulmonary disease and the role of healthy ageing trajectories: A population-based study of middle-aged and older adults.

Journal:	*BMJ Open*
Manuscript ID	bmjopen-2021-050947
Article Type:	Original research
Date Submitted by the Author:	08-Mar-2021
Complete List of Authors:	Bayes-Marin, Ivet; Parc Sanitari Sant Joan de Déu, Research, Innovation and Teaching Unit; Universitat de Barcelona Facultat de Medicina<br>Sanchez-Niubo, Albert ; Parc Sanitari Sant Joan de Deu, ; Instituto de Salud Carlos III, Centro de Investigación Biomédica en Red de Salud Mental (CIBERSAM)<br>Fernández, Daniel; Polytechnic University of Catalonia, Serra Húnter fellow, Department of Statistics and Operations Research<br>Haro, Josep; Parc Sanitari Sant Joan de Deu, Research, Innovation and Teaching Unit; Centro de Investigación Biomédica en Red de Salud Mental<br>Olaya, Beatriz; Parc Sanitari Sant Joan de Deu, Research, Innovation and Teaching Unit; Centro de Investigación Biomédica en Red de Salud Mental
Keywords:	Chronic airways disease < THORACIC MEDICINE, EPIDEMIOLOGY, PUBLIC HEALTH

SCHOLARONE™
Manuscripts

BMJ

I, the Submitting Author has the right to grant and does grant on behalf of all authors of the Work (as defined in the below author licence), an exclusive licence and/or a non-exclusive licence for contributions from authors who are: i) UK Crown employees; ii) where BMJ has agreed a CC-BY licence shall apply, and/or iii) in accordance with the terms applicable for US Federal Government officers or employees acting as part of their official duties; on a worldwide, perpetual, irrevocable, royalty-free basis to BMJ Publishing Group Ltd ("BMJ") its licensees and where the relevant Journal is co-owned by BMJ to the co-owners of the Journal, to publish the Work in this journal and any other BMJ products and to exploit all rights, as set out in our licence.

The Submitting Author accepts and understands that any supply made under these terms is made by BMJ to the Submitting Author unless you are acting as an employee on behalf of your employer or a postgraduate student of an affiliated institution which is paying any applicable article publishing charge ("APC") for Open Access articles. Where the Submitting Author wishes to make the Work available on an Open Access basis (and intends to pay the relevant APC), the terms of reuse of such Open Access shall be governed by a Creative Commons licence – details of these licences and which Creative Commons licence will apply to this Work are set out in our licence referred to above.

Other than as permitted in any relevant BMJ Author's Self Archiving Policies, I confirm this Work has not been accepted for publication elsewhere, is not being considered for publication elsewhere and does not duplicate material already published. I confirm all authors consent to publication of this Work and authorise the granting of this licence.

For peer review only

Risk of all-cause mortality associated with chronic obstructive pulmonary disease and the role of healthy ageing trajectories: A population-based study of middle-aged and older adults.

Ivet Bayes-Marin [a,b,c], Albert Sanchez-Niubo [a,b], Daniel Fernández[d], Josep Maria Haro [a,b,c], and Beatriz Olaya [a,b]

[a]Research, Innovation and Teaching Unit, Parc Sanitari Sant Joan de Déu, Sant Boi de Llobregat, 08830 Barcelona, Spain.

[b]Centro de Investigación Biomédica en Red de Salud Mental (CIBERSAM), Instituto de Salud Carlos III, Madrid 28029, Spain.

[c]Department of Medicine, Universitat de Barcelona, Barcelona 08036, Spain.

[d]Serra Húnter fellow, Department of Statistics and Operations Research, Polytechnic University of Catalonia-BarcelonaTech, 08028 Barcelona, Spain.

***Corresponding author:**

Albert Sanchez- Niubo. Research, Innovation and Teaching Unit, Parc Sanitari Sant Joan de Déu, Dr Antoni Pujadas 42, Sant Boi de Llobregat, Barcelona 08830, Spain. E-mail: albert.sanchez@pssjd.org

Word count: 5840

Abstract

Introduction: Chronic obstructive pulmonary disease (COPD) is a major cause of morbidity and mortality worldwide. With a worsening of the disease, there can be an increase of functional ability limitations, limiting exercise performance and self-care. The study of the mortality risk in COPD and its association with health and functioning would allow identifying those vulnerable sectors of the population and the creation of preventive measures and interventions. The aims

For peer review only - http://bmjopen.bmj.com/site/about/guidelines.xhtml

were to study the risk of all-cause mortality associated with COPD and healthy ageing trajectories (HAT) in three birth cohorts and to determine the moderating role of HAT in the association between COPD and all-cause mortality. **Methods:** The total sample was 28,857 people aged 50+ years from waves 1 to 5 of The Survey of Health, Ageing and Retirement in Europe (SHARE). Analyses were conducted separately in three birth cohorts (>1945, 1936-1945, and ≤1935). Latent class growth analysis was used to classify participants into HAT based on their score on the healthy ageing scale. We performed Aalen additive hazards models to explore the associations between COPD, HAT, and mortality. Interactions between COPD and HAT were also explored. **Results:** Three parallel HAT were found in the three birth cohorts ("low", "medium", and "high" healthy ageing). Participants with COPD had an increased mortality risk, but this effect was no longer significant after adjusting for covariates. The "low" HAT was associated with increased mortality risk in the three sub-samples, although this effect was lower after adjustment. The interaction between COPD and HAT was significant only in the ≤1935 birth cohort, indicating that those with COPD and a "low" trajectory had a greater risk of mortality. **Conclusion:** The healthy ageing scale may be a suitable tool to identify patients at higher risk in order to mitigate disease burden and improve patient's quality of life.

Keywords: chronic obstructive pulmonary disease (COPD), mortality, healthy ageing, Europe, population-based study

Strengths and limitations of this study

- The analyses were performed in different birth cohorts (>1945, 1936-1945, and ≤1935) to asses if there were differences in mortality risks that could be related to societal changes, such as trends in lifestyle behaviors and occupation.
- We used a novel measurement scale of healthy ageing including several variables related to intrinsic capacity and functional ability.

For peer review only

- The calculation of Aalen additive hazards models rather than Cox models allowed the inclusion of time-variant variables in the analyses.
- Due to the high percentage of missingness in the age of the diseases diagnosis, we selected the age of the earliest diagnosis of each disease within the five waves.
- For the survival analysis we used the age of the participants instead of the years of the interview, for better interpretation. However, this introduces a problem of left truncation since the age range observed for each participant is different, although we took this into account in the additive regression model.

Introduction

Chronic obstructive pulmonary disease (COPD) is a major cause of morbidity and mortality worldwide[1,2]. COPD is expected to become the third leading cause of death by 2030[3]. The growing burden of COPD is a consequence of the population ageing and the continued use of tobacco, which is considered its main risk factor[4]. Moreover, air pollution has been associated with acute exacerbations of COPD, increased respiratory morbidity and mortality[5].

COPD is characterized by a progressive airflow limitation associated with an abnormal inflammatory response of the lungs to noxious particles or gases[6]. With a worsening of the disease, there can be an increase of functional ability limitations in the activities of daily living (ADL), and in the instrumental activities of daily living (IADL), limiting exercise performance and self-care[7–9]. COPD has increasingly been recognized as a multi-component disease, associated with a wide range of physical diseases and psychological disorders[10]. Non-communicable diseases (NCDs) such as hypertension, cardiovascular diseases, diabetes, cancer, and depression commonly co-exist in COPD patients, worsening its progression[1,2,11]. Furthermore, cognitive impairment is common among COPD patients, suggesting that impaired performance in neuropsychological tests might be a predictor of early mortality for people diagnosed with COPD[10].

For peer review only - http://bmjopen.bmj.com/site/about/guidelines.xhtml

For peer review only

Despite being a growing public health concern, there is a lack of epidemiological data about the prevalence and distribution of COPD[6,12]. The paucity of information on COPD prevalence and incidence is partly due to differences in the methods used for its diagnosis and classification, often being underestimated[6]. These differences in the assessment methods and definitions have also hampered the comparison of COPD prevalence and impact across countries. Both The Burden of Lung Disease (BOLD) project[4] and the Latin American Project for the Investigation of Obstructive Lung Disease (PLATINO)[13] were developed to map the COPD prevalence using the same methodology in different countries. Those studies were performed in China and Turkey, and five Latin American countries, respectively. Nevertheless, there is a lack of data on the prevalence and distribution of COPD in Europe[2,14]. Additionally, available studies suggest large differences across European countries in terms of prevalence rates of COPD and the associated death rates. In a systematic review, COPD prevalence ranged from 3% in Finnish women to 57% in Italian men and women[15]. Some differences have also been found in COPD-related mortality across European countries and between men and women. Overall, regarding European countries, COPD-related mortality rates appeared to decline in men in most countries from 1995 to 2017, whereas mortality rates due to COPD increased in women from +2% per year in Austria to +4.2% or +4.8% per year in the Czech Republic and Hungary, respectively[16].

In that sense, a longitudinal integrated dataset that considers different European countries could be particularly useful in the study of the risk of mortality associated with COPD in different European countries. The study of the mortality risk in COPD patients, as well as its association with several variables related to health and functioning, would allow identifying those vulnerable sectors of the population and the creation of preventive measures and interventions in diverse healthcare systems.

Previous studies focused on the association between exercise capacity and mortality among COPD patients, which has been considered one of the best predictors of mortality[17–19].

For peer review only - http://bmjopen.bmj.com/site/about/guidelines.xhtml

Measures of exercise capacity include indicators such as body mass index, airflow obstruction, dyspnea, handgrip strength, and the sit-to-stand test[20]. Nevertheless, these indicators of exercise capacity are just measures of intrinsic capacity that do not capture the individual's functional ability over the life-course. In that sense, the functional ability is the result of the interaction of the individuals' intrinsic capacity, including physical and mental capacities; and their environment, as access to medications, personal and assistive support, or physical barriers[21]. Therefore, a measure assessing both intrinsic capacity and functional ability may be a better way to capture a person's healthy aging.

Several authors advocate for using composite measures as the International Classification of Functioning, Disability, and Health to assess COPD patients' complexity, including also functional capacity and functional performance[22]. Related to this, the Ageing Trajectories of Health: Longitudinal Opportunities and Synergies (ATHLOS) project[23] developed a healthy ageing scale[21] using 16 international cohort studies to determine the intrinsic capacity and functional ability of the participants allowing comparisons across countries. The healthy ageing scale is composed of several domains such as vitality, sensory skills, locomotion/mobility, cognition, ADL, and IADL. Thus, this measure includes not only measures of exercise capacity, but also functionality that could be affected by the course of COPD disease and impact on the patients' quality of life.

The aims of the present paper are: 1) to study the risk of all-cause mortality associated with COPD and healthy ageing trajectories (HAT) in three population-based cohorts of middle-aged and older adults; 2) to determine the moderating role of HAT in the association between COPD and all-cause mortality. We speculated that a HAT characterized by low levels of healthy ageing would be significantly associated with an increased risk of mortality in people with COPD, whereas individuals with higher levels of healthy ageing and COPD would have a lower risk of mortality.

For peer review only - http://bmjopen.bmj.com/site/about/guidelines.xhtml

Methods

Study design and Data Collection

The present study used data from five waves of The Survey of Health, Ageing and Retirement in Europe (SHARE)[24]. SHARE is a multidisciplinary, cross-national panel database that contains a broad range of information on health, socioeconomic status, and social networks of European citizens aged 50 and older. The first wave took place in 2004-2005, constituted by more than 22,000 persons born in 1954 and earlier, and the following waves were conducted approximately every two years. The interviewers used computer-assisted personal interviewing (CAPI) to collect most of the data in all waves. Additionally, in waves 1, 2, and 4, self-administered questionnaires were handed out after the CAPI completion. If a respondent passed away during the study, then an end-of-life interview was conducted with a proxy.

The overall individual response rate at baseline was 60.1% and the wave-to-wave retention rate of participants from wave 1 was higher than 55% in all the countries[25]. All participants gave written consent. Ethical approvals for waves from 1 to 3 were granted by the Ethics Committee of the University of Mannheim[24]. For waves 4 and 5, the SHARE projects were reviewed and approved by the Ethics Council of the Max-Planck Society[26]. Further details concerning the study design of SHARE can be found elsewhere[24].

The following countries were included in the present analysis: Denmark, Sweden, Greece, Italy, Spain, Israel, Austria, Belgium, France, Germany, Netherlands, and Switzerland. We excluded those participants who were incorporated in the subsequent waves due to the sample's refreshments (*n*=30,816). The analyses focused on people aged 50 years and older who completed a non-proxy interview at baseline, resulting in an analytical sample of 28,857 respondents.

For peer review only

Patient and public involvement

No patient involved.

Measurements

- *All-cause mortality*

The death of a participant was confirmed by interviewing a proxy-respondent since information on the deceased was not linked to national death registries[25,27]. If confirmed, the date of death was obtained from end-of-life interviews with a proxy respondent[25,27]. Participants were characterized as survivors or censored if they were alive at the end of the study period, and dead if they died during the study period.

Survival time was calculated in years and as follows: 1) from baseline to the reported date of death or the final assessment date for those participants who were alive at the end of 2013; or 2) in the case that a participant reported being diagnosed with COPD at baseline, survival time was calculated from baseline. Besides, for the set of patients who reported a new diagnosis of COPD during the follow-up period, we considered the first time of the observation as the age at which they were newly diagnosed.

- *Chronic obstructive pulmonary disease (COPD)*

Participants reported whether a doctor ever informed them that they had "COPD such as chronic bronchitis or emphysema". In the present study, we considered the first age in which a participant reported having been diagnosed with COPD instead of considering the presence/absence of COPD at baseline because the participant might be diagnosed in the subsequent waves. Therefore, COPD diagnosis was treated as a time-variant variable.

- *Healthy ageing scale*

We used an international scale of healthy ageing measurement developed by the ATHLOS consortium[21,28]. This scale used items about intrinsic capacity and functional ability based on

For peer review only - http://bmjopen.bmj.com/site/about/guidelines.xhtml

the World Mental Health's (WHO) concept of healthy ageing[29]. The healthy ageing scale covers different domains, such as vitality, sensory skills, locomotion/mobility, cognition, ADL, and IADL. Thirty-nine study-specific variables were harmonised into dichotomous items indicating the presence or absence of difficulties (see Supplementary Table 1). Final scores were estimated for all individuals and converted to *T*-scores with a mean of 50 and a standard deviation of 10. In our study, we applied latent class growth analysis (LCGA)[30] to identify longitudinal trajectories according to the healthy ageing scale score across the waves and classify the participants into those trajectories.

- *Covariates*

Demographic variables included sex (male/female), age (in years), level of education (less than primary, primary, secondary, and tertiary), marital status (single, married or currently cohabiting, separated or divorced, and widowed), and quintiles of household wealth (first quintile indicating lowest level).

Lifestyles and health behaviors included ever smoked and practice of vigorous physical activity during the last two weeks and were coded as *yes* or *no*. The following self-reported diagnoses of NCDs different from COPD were included: diabetes, hypertension, joint disorders (arthritis, rheumatism, or osteoarthritis), asthma, myocardial infarction, and stroke. Similar to COPD, we selected the age of the earliest diagnosis of each NCD across the five waves, considering them as time-variant variables.

Depression was assessed with the EURO-D 12-item scale, which was developed and validated for the EURODEP studies to measure depressive symptoms across European countries accounting for regional differences[31,32]. The EURO-D score ranges from 0 to 12, with higher scores meaning higher levels of depression, being 4 or greater than the proposed cut-off score that has been selected to create a dichotomous depression variable (yes/no)[31].

For peer review only - http://bmjopen.bmj.com/site/about/guidelines.xhtml

For peer review only

Finally, we grouped the countries into 3 European regions according to the World Health Organization (WHO) and the United Nations Statistical Division (UNSD) regional classification[33,34]. Thus, Northern Europe was constituted by Denmark and Sweden, Western Europe included Austria, Belgium, France, Germany, Israel, the Netherlands, and Switzerland, and Southern Europe included Spain, Italy, and Greece.

Statistical Analyses

We divided the sample into three groups according to the year of birth of the participants and keeping proportional sample sizes. The first group (*n*=9,866) was composed of those participants who were born after 1945 (the youngest participants: aged 50+), the second group (*n*=9,254) comprised participants born between 1936 and 1945 (ages from 58 to 70 years old), and the third one (*n*=9,739) encompassed individuals who were born in 1935 or earlier (the oldest participants: from 69 to 104 years old). Analyses were independently conducted in these three birth cohorts.

Latent class growth analysis (LCGA) was used to classify individuals into trajectories based on their score on the healthy ageing scale[30]. The number of trajectories was determined by analyzing group models from 1 to 5 trajectories. The optimal model was selected according to the Bayesian information criterion, where the lowest value indicates the better fit[35,36], and the sample size of the trajectory group; a sample size lower than 5% was considered insufficient to identify classes[36].

To avoid the assumption of proportionality of the Cox regression hazards, we conducted an Aalen additive hazards modeling approach, which explores the associations between COPD and time to death[37,38]. These models can provide a better picture of how the effects of covariates develop over time without assuming the proportional risk hypothesis as in the Cox regression models[39]. Parameters of these models are arbitrary cumulative regression functions that represent the cumulative excess risk at each unit of time and are useful to assess changes over

For peer review only - http://bmjopen.bmj.com/site/about/guidelines.xhtml

time graphically[40]. Confidence intervals above zero for a concrete age indicate a significant risk, below zero indicate a protective effect, and confidence intervals including zero show a non-significant risk[41]. Models were adjusted for sex, age, marital status, level of education, household wealth, region, vigorous physical activity, tobacco consumption, HAT, depression, and presence of NCDs, all as time-varying covariates. The interaction between COPD and HAT was also assessed. Age was used as the time measure. Participants who were alive at the end of the study period or in their final assessment were censored. In the modeling process, we also took into account that the data were left-truncated because we considered the first interview as the time of diagnosis in the case of the participants who had been diagnosed by an NCD before baseline. All analyses were performed using R Version 4.0.3.[42]. Statistical significance was set at $p<0.05$.

Results

We identified three HAT in each of the three birth cohorts according to lower BIC and the sample sizes not lower than 5% (Supplementary Table 2 and Supplementary Figure 1). Although in the oldest birth cohort 4 trajectories met the selection criteria, we decided to select a three-trajectory model to facilitate comparison between the three cohorts. In all birth cohorts, the trajectories were parallel. The first trajectory group included individuals with the highest scores on the healthy ageing scale and the third those with the worse scores. We named each trajectory group as “high”, “medium”, and “low”, respectively.

Table 1 shows the characteristics of participants. Those participants of the oldest group (born ≤1935) showed a higher prevalence of COPD (12.50%), followed by those born between 1935 and 1945 (9.57%), ($p<0.001$). The oldest group presented lower proportions of the “high” HAT (31.60%), compared with the other two birth cohorts ($p<0.001$). Finally, the proportion of deaths increased with age, being lower in the >1945 (2.07%) and higher in the ≤1935 sub-sample (16.90%) ($p<0.001$).

For peer review only - http://bmjopen.bmj.com/site/about/guidelines.xhtml

Table 1. Main characteristics of the sample broken down by year of birth

Characteristics	Years of birth cohort			*p* value[1]
	≤1935 (N=9738)	1936-1945 (N=9254)	>1945 (N=9865)	
**Female, n (%)**	5407 (55.50)	4879 (52.70)	5382 (54.60)	<0.001
**Age, mean (SD)**	74.40 (5.94)	61.30 (2.95)	52.10 (2.58)	<0.001
**Marital status, n (%)**				<0.001
Single	452 (4.64)	468 (5.06)	638 (6.47)	
Married	5832 (59.90)	7343 (79.30)	8051 (81.60)	
Divorced	338 (3.47)	603 (6.52)	950 (9.63)	
Widowed	3646 (37.40)	1203 (13.00)	531 (5.38)	
**Education level, n (%)**				<0.001
Less than primary	994 (10.20)	417 (4.51)	246 (2.49)	
Primary	3895 (40.00)	2513 (27.20)	1604 (16.30)	
Secondary	3734 (38.30)	4570 (49.40)	5520 (56.00)	
Tertiary	1115 (11.40)	1754 (19.00)	2495 (25.30)	
**Wealth quintiles, n (%)**				<0.001
1st (worst)	1798 (18.50)	801 (8.66)	687 (6.96)	
2nd	2479 (25.50)	1380 (14.90)	960 (9.73)	
3rd	2185 (22.40)	1895 (20.50)	1481 (15.00)	
4th	1721 (17.70)	2247 (24.30)	2494 (25.30)	
5th (best)	1555 (16.00)	2931 (31.70)	4243 (43.00)	
**Region, n (%)**				0.049
Northern Europe	1557 (16.00)	1483 (16.00)	1473 (14.90)	
Western Europe	4842 (49.70)	4676 (50.50)	5090 (51.60)	
Southern Europe	3339 (34.30)	3095 (33.40)	3302 (33.50)	
**Healthy ageing trajectories, n (%)**				<0.001
High	3073 (31.60)	4620 (49.90)	5962 (60.40)	
Medium	4713 (48.40)	3491 (37.70)	3050 (30.90)	
Low	1952 (20.00)	1143 (12.40)	853 (8.65)	
**Physical activity, n (%)**	4934 (50.70)	7107 (76.80)	8283 (84.00)	<0.001
**Ever smoked, n (%)**	3910 (40.20)	4572 (49.40)	5543 (56.20)	<0.001
**Diseases, n (%)**				
Diabetes	1821 (18.70)	1628 (17.60)	1132 (11.50)	<0.001
Hypertension	5334 (54.80)	4647 (50.20)	3500 (35.50)	<0.001
Joint disorders	3914 (40.20)	3018 (32.60)	2407 (24.40)	<0.001
Asthma	703 (7.22)	550 (5.94)	497 (5.04)	<0.001
COPD	1214 (12.50)	886 (9.57)	622 (6.31)	<0.001
Myocardial infarction	3022 (31.00)	1751 (18.90)	957 (9.70)	<0.001
Stroke	1119 (11.50)	565 (6.11)	332 (3.37)	<0.001
Depression	4340 (44.60)	3387 (36.60)	3444 (34.90)	<0.001
**Death, n (%)**	1642 (16.90)	451 (4.87)	204 (2.07)	<0.001

Note. Household income was divided into 5 quintiles (the first indicating the lowest income). Marital status "married" category included "currently married or cohabiting", and "divorced" included "divorced or separated". Abbreviations: COPD, chronic obstructive pulmonary disease. [1]Based on T-tests for numerical variables and Chi-square tests for categorical variables.

For peer review only - http://bmjopen.bmj.com/site/about/guidelines.xhtml

Three Aalen regression models were conducted: one with only the variable COPD, the second with only the variable of HAT, and the third with COPD and the HAT adjusted for covariates. The estimated cumulative coefficients of the first and second model are presented in Supplementary Figure 2, and those from the third model are presented in Figure 1, according to the >1945, 1936-1945, and ≤1935 sub-samples, respectively. In the first model, COPD diagnosis had a significant risk on mortality in the three birth cohort groups: from 74 years old onwards in the ≤1935 sub-sample, from 65 years old onwards in the other two sub-samples (see Supplementary Figure 2). In the second model, regarding the HAT, those individuals classified in "low" trajectories had a significant risk of mortality: in the ≤1935 sub-sample there was a significant risk of death from 76 to 94 years, and from 97 to 98; in the 1936-1945 sub-sample from 63 onwards; and in the >1945 sub-sample from 60 onwards. Those following a "medium" HAT had a significant risk of death in the ≤1935 sub-sample intermittently from 76 to 86 years and at 98 years; in the 1936-1945 sub-sample from 65 onwards; and in the >1945 sub-sample from 62 onwards (see Supplementary Figure 2).

Figure 1 shows the estimated cumulative coefficients calculated from the third model (including all variables) for each birth cohort. In this model, although the risk effect of COPD increases across age, it was rather non-significant (only a small effect in the ≤1935 sub-sample around 76 and 77 years old). In the case of the HAT, "low" trajectories were associated with a higher risk of mortality in the case of the ≤1935 sub-sample (from 88 to 90). There was a significant mortality risk in the 1936-1945 and the >1945 birth cohorts (from 71 onwards and 60 onwards, respectively). "Medium" HAT had only a significant effect in the 1936-1945 sub-sample, from 74 onwards. The interaction between COPD and HAT was assessed in the third model. A significant effect was only found in the model with the ≤1935 sub-sample. The interaction showed that there was a significant effect (higher risk of death) for participants with COPD and a "low" HAT, with the highest risk of death at the age of 75 and from 81 to 87.

For peer review only - http://bmjopen.bmj.com/site/about/guidelines.xhtml

Completed and detailed results of the fitting of Aalen's additive regression models are presented in Supplementary Figure 3.

Discussion

We analysed the association of COPD with the risk of mortality and the moderating role of HAT in the SHARE study, a population-based cohort of middle-aged and older adults from 12 European countries who were followed up for 9 years. With the aim of accounting for potential cohort effects, we analysed the results separately in three groups: those born after 1945 (aged 50+), born between 1936 and 1945 (ages from 58 to 70 years old), and born in 1935 or earlier (ages from 69 to 104 years old).

Our findings show that COPD increased the risk of mortality in the three birth cohorts. However, this association was no longer significant after adjusting for demographic and economic variables, presence of other NCDs and depression, and HAT. In line with previous research, the study of mortality in COPD patients is quite cumbersome, and multiple variables may play a role in this association. For example, lung cancer and COPD mortality were assessed including several variables (residential characteristics, marital status, education, health insurance, and family income) in a research study based on The National Longitudinal Mortality Study in the United States[43]. They found that COPD mortality rates were highest among 65 to 74 years old, in males and non-Hispanic whites[43]. The results concerning the periods are consistent with those we found before adjustment, suggesting the existence of a period of increased risk of mortality in COPD patients. In another study based on The National Mortality Database of Statistics Canada, the mortality related to COPD varied by age, sex, birth cohort, and the province[44]. In that study, the mortality risk attributed to COPD decreased in male and female cohorts born after 1920 to 1924, whereas between 1971 and 1983 the mortality ratios were stable[44]. Thus, performing the analyses considering different birth cohorts seems to be appropriate, since

For peer review only - http://bmjopen.bmj.com/site/about/guidelines.xhtml

exposure to risk factors for COPD such as tobacco consumption or occupational pollution might greatly vary across birth cohorts. Moreover, previous studies on the risk of COPD mortality have reported differences in terms of age, sex, birth cohort, location, household income, education, and marital status[43–46]. Thus, the study of mortality associated with COPD needs to account for the potential confounding effects of these risk factors.

One potential confounder is the region of residence, as indicated in previous studies[47]. Despite not being the focus of our study, we identify that living in Western or Southern Europe had a protective effect on the risk of all-cause mortality, compared to Northern Europe (Denmark and Sweden). Similarly, Blanco et al (2017), found a lower mean COPD prevalence in Southern Europe (10.8%) compared to Northern Europe (11,5%), although variations in terms of COPD prevalence were also found among countries of the same European region[12]. In Northern Europe, it was higher in Denmark (ranging from 12% to 25%) than in Sweden (ranging from 2% to 20%); whereas in Southern Europe, Italy showed higher prevalence (ranging from 12% to 23%), than in Spain (from 7 to 10%)[12]. The greater COPD prevalence and its associated mortality risk in Denmark could be a consequence of a very high smoking prevalence in the past 5 decades, resulting in the highest COPD prevalence in the western world[48]. This heterogeneity among countries and regions might suggest the need for a better understanding of the underlying mechanisms.

Regarding the HAT, our results seem to confirm that participants (from different birth cohorts) with "low" and "medium" HAT (i.e, worse health status) have a higher risk of mortality, compared to those classified into "high" HAT. This effect remains after adjusting for covariates although in the case of the "medium" trajectories only a significant effect was found in the 1936-1945 birth cohort (constituted by people aged 58 to 70 years old). According to our results, "low" trajectories seem to discriminate in a better way a poorer health status and to predict mortality, even after adjusting for confounders. Previous studies examined the connection between

For peer review only - http://bmjopen.bmj.com/site/about/guidelines.xhtml

healthy ageing and mortality, albeit using different indicators[49,50]. In a South Brazilian population-based cohort, researchers differentiated between normal ageing and successful ageing (defined as a very good state of health, a complete absence of functional disability and mood changes, and no cognitive impairment)[49]. They detected that successful agers had lower mortality rates, and the normal agers had a higher risk for mortality[49]. These results may be extrapolated to our "low" and "high" HAT, being the last the equivalent to "successful ageing". Another study used The Healthy Ageing Index (HAI) as a summary measure of physiologic aging[50], composed of markers of cardiovascular, lung, cognitive, metabolic, and kidney function. In that study, HAI scores tended to increase with age (meaning worse healthy ageing) and predicted mortality from a given time-point[50]. Hence, composite measures of ageing seem to be powerful tools to predict mortality and to identify individuals at a higher risk.

One of the main results from our study is that the association between COPD and risk of mortality depended upon the HAT of the oldest participants (i.e., born ≤1935). Individuals with COPD and a "low" trajectory of healthy ageing were more likely to die at the age of 75 years old and from 81 to 87, compared with people with COPD and a "medium" or "high" HAT. The healthy ageing scale covers several domains (vitality, sensory skills, mobility, cognition, and ADL/IADL) and could be negatively affected in those patients with worse COPD symptoms[7–10]. The fact that these results were found only in the oldest sub-sample may be related to the course of the disease since COPD is a progressive disease and exacerbations and hospitalizations are particularly common among older individuals[51]. Our results point out temporary spaces where older COPD patients with a "low" HAT are at higher risk of mortality. Thus, future efforts should be concentrated on those aged 75 years old and from 81 to 87.

To the best of our knowledge, few studies have analysed the relationship between health status in COPD patients. These studies were based on self-reported perceived health status assessed through the SF-12 questionnaire, which is a generic instrument to evaluate physical and mental

For peer review only - http://bmjopen.bmj.com/site/about/guidelines.xhtml

health[52,53]. The main finding in one of these studies, that used data from the BOLD project, was that COPD severity was an important determinant of health status (more severity linked to poorer health status)[52]. Although these studies considered the health status of people with COPD, we have not found any study that used a composite measure of healthy ageing as we have done. An integrated measure assessing intrinsic capacity and functional ability could be a useful tool in daily clinical practice for patient prognosis, as well as a mortality predictor, and for the creation of future public health strategies addressing COPD patients' needs[21]. While it is true that other composite tools to predict COPD mortality are available (such as St George's Respiratory Questionnaire[54], or the BODE index[55]), the healthy ageing scale is a comprehensive tool that could be applied not only to COPD patients but also to patients with multimorbidity.

Strengths and limitations

These findings should be interpreted in light of the following limitations. Firstly, the presence or absence of COPD and NCDs was based on self-reported diagnostics, thus they might be affected by measurement errors. Nevertheless, some authors sustain self-reported diagnostics as a well-established method for the measurement of NCDs in population-based studies[56]. Secondly, we did some assumptions in terms of age of diagnosis. Due to the high percentage of missingness (48%) in the age of the NCD diagnosis, we selected the age of the earliest diagnosis of each NCD within the five waves. That is, we coded the age of the participant in the wave he/she reported the first time having some of the included diseases. Despite being an assumption, there are only two years between each wave in the SHARE study. Thus, we believe that there is not a huge impact on our conclusions. Thirdly, we split the sample into three birth cohorts when performing the analyses, and we reported the mortality risk in each group. By doing so, we captured potential cohort effects which people from different birth cohorts can be influenced by different exposure to COPD-related risk factors that contribute differently to mortality, as the different trends in smoking prevalence. For each birth cohort, the survival

For peer review only - http://bmjopen.bmj.com/site/about/guidelines.xhtml

analysis can be focused according to the years of the interview or according to the age of the participants. We finally decided to do it according to the age of the participants because working with time-varying variables and without the assumption of proportional risks, the fluctuations in mortality risk according to age could be better interpreted. However, this introduces a problem of left truncation since the age range observed for each participant is different, although we took this into account in the additive regression model. Fourthly, another issue is that the age range observed for each birth cohort is also different so that the excess cumulative risk curve starts at the first observed age. Therefore, the bias of the healthy participant in the first wave of the study means that in the first ages of observation there is no significant excess risk.

Alongside these limitations, this study had a number of strengths. Firstly, the analyses were performed in different birth cohorts (>1945, 1936-1945, and ≤1935) to assess if there were differences in mortality risks that could be related to societal changes, such as trends in lifestyle behaviours and occupation. Secondly, we used a novel measurement scale of healthy ageing including several variables related to intrinsic capacity and functional ability. Compared with the use of different indicators of health separately, we believe that using an integrated and reliable measure of health status is a powerful tool to predict the mortality risk of the participants. Thirdly, the calculation of Aalen additive hazards models rather than Cox models allowed the inclusion of time-variant variables in the analyses.

Conclusion

COPD is a costly and preventable disease that has large-scale implications for patients' quality of life and society in general[57,58]. Our findings suggest that the association between COPD and risk of mortality in the general population of middle-aged and older adults might be explained by the presence of other risk factors. However, for older people with COPD (i.e., aged 69 or older), having a poor trajectory of healthy ageing might compromise their survival. Especial

For peer review only - http://bmjopen.bmj.com/site/about/guidelines.xhtml

attention should be paid to these patients, with the healthy ageing scale as a potential suitable tool to do identify older patients with COPD at high risk of mortality[20].

Competing interests

The authors declare no conflict of interest.

Patient consent for publication

Not required.

Data sharing statement: The original data of the Survey of Health, Ageing and Retirement in Europe – SHARE is available on the official website (http://www.share-project.org/home0.html). R codes for harmonizing the healthy ageing scale is available on https://athlos.pssjd.org/study/share-hs.

Funding

This work was supported by the five-year Ageing Trajectories of Health: Longitudinal Opportunities and Synergies (ATHLOS) project. The ATHLOS project has received funding from the European Union's Horizon 2020 research and innovation program under grant agreement No 635316. BO's work is supported by the Miguel Servet program (CP20/00040), funded by Instituto de Salud Carlos III and co-funded by European Union (ERDF/ESF, "Investing in your future"). DF's work has been supported by Marsden grant E2987-3648 administrated by the Royal Society of New Zealand) and by grant 2017 SGR 622 (GRBIO) administrated by the Departament d'Economia i Coneixement de la Generalitat de Catalunya (Spain).

Acknowledgments

The SHARE study is funded by the European Commission through FP5 (QLK6-CT-2001-00360), FP6 (SHARE-I3: RII-CT-2006-062193, COMPARE: CIT5-CT-2005-028857, SHARELIFE: CIT4-CT-2006-028812) and FP7 (SHARE-PREP: N°211909, SHARE-LEAP: N°227822, SHARE M4: N°261982). Additional funding from the German Ministry of Education and Research, the Max Planck Society

For peer review only - http://bmjopen.bmj.com/site/about/guidelines.xhtml

for the Advancement of Science, the U.S. National Institute on Aging (U01_AG09740-13S2, P01_AG005842, P01_AG08291, P30_AG12815, R21_AG025169, Y1-AG-4553-01, IAG_BSR06-11, OGHA_04-064, HHSN271201300071C) and from various national funding sources is gratefully acknowledged (see www.share-project.org).

Author contributions

IB: Participated in the database management, drafted the paper, carried out the statistical analyses and worked on the interpretation of data. She also gave final approval of the version to be published and agreed to be accountable for all aspects of the work in ensuring that questions related to the accuracy or integrity of any part of the work are appropriately investigated and resolved; **AS:** Participated in the study design, database management, carried out the statistical analyses, gave statistical support and critical revision of the paper. He also gave final approval of the version to be published and agreed to be accountable for all aspects of the work in ensuring that questions related to the accuracy or integrity of any part of the work are appropriately investigated and resolved; **DF:** Participated in the statistical support and critical revision of the paper. He also gave final approval of the version to be published and agreed to be accountable for all aspects of the work in ensuring that questions related to the accuracy or integrity of any part of the work are appropriately investigated and resolved; **JMH:** Participated in the acquisition of data, and critical revision of the paper. He also gave final approval of the version to be published and agreed to be accountable for all aspects of the work in ensuring that questions related to the accuracy or integrity of any part of the work are appropriately investigated and resolved; **BO:** Participated in the critical revision of the paper. She also gave final approval of the version to be published and agreed to be accountable for all aspects of the work in ensuring that questions related to the accuracy or integrity of any part of the work are appropriately investigated and resolved.

References

1 Miniati M, Monti S, Pavlickova I, *et al.* Survival in COPD: impact of lung dysfunction and

comorbidities. *Med* 2014;**93**:e76. doi:10.1097/MD.0000000000000076

2 Okely JA, Shaheen SO, Weiss A, *et al.* Wellbeing and chronic lung disease incidence: the Survey of Health, Ageing and Retirement in Europe. *PLoS One* 2017;**12**:e0181320. doi:10.1371/journal.pone.0181320

3 World Health Organization. World Health Statistics 2008. 2008. https://www.who.int/whosis/whostat/EN_WHS08_Full.pdf?ua=1 (accessed 20 May 2020).

4 Buist AS, McBurnie MA, Vollmer WM, *et al.* International variation in the prevalence of COPD (The BOLD Study): a population-based prevalence study. *Lancet* 2007;**370**:741–50. doi:10.1016/S0140-6736(07)61377-4

5 Duan R-R, Hao K, Yang T. Air pollution and chronic obstructive pulmonary disease. *Chronic Dis Transl Med* 2020;**6**:260–9. doi:10.1016/j.cdtm.2020.05.004

6 Rosenberg SR, Kalhan R, Mannino DM. Epidemiology of chronic obstructive pulmonary disease: prevalence, morbidity, mortality, and risk factors. *Semin Respir Crit Care Med* 2015;**36**:457–69. doi:10.1055/s-0035-1555607

7 Bourbeau J. Activities of Life: the COPD patient. *COPD* 2009;**6**:192–200. doi:10.1080/15412550902902638

8 Barusso MS, Gianjoppe-Santos J, Basso-Vanelli RP, *et al.* Limitation of activities of daily living and quality of life based on COPD combined classification. *Respir Care* 2015;**60**:388–98. doi:10.4187/RESPCARE.03202

9 Medina-Mirapeix F, Bernabeu-Mora R, Piedad Sánchez-Martínez M, *et al.* Mobility limitations related to reduced pulmonary function among aging people with chronic obstructive pulmonary disease. *PLoS One* 2018;**13**:e0196152. doi:10.1371/journal.pone.0196152

10 Dodd JW. Lung disease as a determinant of cognitive decline and dementia. *Alzheimer's Res Ther* 2015;**7**:1–8. doi:10.1186/s13195-015-0116-3

11 Eroglu SA, Gunen H, Yakar HI, *et al.* Influence of comorbidities in long-term survival of chronic obstructive pulmonary disease patients. *J Thorac Dis* 2019;**11**:1379–86. doi:10.21037/jtd.2019.03.78

12 Blanco I, Diego I, Bueno P, *et al.* Geographical distribution of COPD prevalence in Europe, estimated by an inverse distance weighting interpolation technique. *Int J Chron Obs Pulmon Dis* 2017;**13**:57–67. doi:10.2147/COPD.S150853

13 Menezes AMB, Perez-Padilla R, Jardim JRB, *et al.* Chronic obstructive pulmonary disease in five Latin American cities (the PLATINO study): A prevalence study. *Lancet* 2005;**366**:1875–81. doi:10.1016/S0140-6736(05)67632-5

14 Blanco I, Diego I, Bueno P, *et al.* Geographical distribution of COPD prevalence in Europe, estimated by an inverse distance weighting interpolation technique. *Int J Chron Obs Pulmon Dis* 2017;**13**:57–67. doi:10.2147/COPD.S150853

15 Nowak D, Berger K, Lippert B, *et al.* Epidemiology and health economics of COPD across Europe: A critical analysis. Treat. Respir. Med. 2005;**4**:381–95. doi:10.2165/00151829-200504060-00003

16 Lortet-Tieulent J, Soerjomataram I, López-Campos JL, *et al.* International trends in COPD mortality, 1995-2017. *Eur Respir J* 2019;**54**:1901791. doi:10.1183/13993003.01791-

For peer review only - http://bmjopen.bmj.com/site/about/guidelines.xhtml

2019

17 Pinto-Plata VM, Cote C, Cabral H, *et al.* The 6-min walk distance: Change over time and value as a predictor of survival in severe COPD. *Eur Respir J* 2004;**23**:28–33. doi:10.1183/09031936.03.00034603

18 Spruit MA, Polkey MI, Celli B, *et al.* Predicting Outcomes from 6-Minute Walk Distance in Chronic Obstructive Pulmonary Disease. *J Am Med Dir Assoc* 2012;**13**:291–7. doi:10.1016/j.jamda.2011.06.009

19 Puhan MA, Garcia-Aymerich J, Frey M, *et al.* Expansion of the prognostic assessment of patients with chronic obstructive pulmonary disease: the updated BODE index and the ADO index. *Lancet* 2009;**374**:704–11. doi:10.1016/S0140-6736(09)61301-5

20 Puhan MA, Siebeling L, Zoller M, *et al.* Simple functional performance tests and mortality in COPD. *Eur Respir J* 2013;**42**:956–63. doi:10.1183/09031936.00131612

21 Sanchez-Niubo A, Forero CG, Wu Y-T, *et al.* Development of a common scale for measuring healthy ageing across the world: results from the ATHLOS consortium. *Int J Epidemiol* 2020;**dyaa236**:1–13. doi:10.1093/ije/dyaa236

22 Bui KL, Nyberg A, Maltais F, *et al.* Functional tests in chronic obstructive pulmonary disease, Part 1: Clinical relevance and links to the international classification of functioning, disability, and health. In: *Annals of the American Thoracic Society*. American Thoracic Society 2017. 778–84. doi:10.1513/AnnalsATS.201609-733AS

23 Sanchez-Niubo A, Egea-Cortés L, Olaya B, *et al.* Cohort profile: the ageing trajectories of health - longitudinal ppportunities and synergies (ATHLOS) project. *Int J Epidemiol* 2019;**48**:1052-1053i. doi:org/10.1093/ije/dyz077

24 Börsch-Supan A, Brandt M, Hunkler C, *et al.* Data resource profile: The survey of health, ageing and retirement in europe (SHARE). *Int J Epidemiol* 2013;**42**:992–1001. doi:10.1093/ije/dyt088

25 Bergmann, Michael; Kneip, Thorsten; De Luca, Giuseppe; Scherpenzeel A. Survey Participation in the Survey of Health, Ageing and Retirement in Europe (SHARE), Wave 1-6. Based on Release 6.0.0 (March 2017). Munich: Munich Center for the Economics of Aging (MEA): 2017.

26 Wolfrum R. Opinion of the ethics council of the Max Planck society on the "SHARE" project. 2016.http://www.share-project.org/fileadmin/pdf_documentation/SHARE_ethics_approvals.pdf

27 Bergmann, M. Kneip, T., De Luca, G., & Scherpenzeel A. Survey participation in the Survey of Health, Ageing and Retirement in Europe (SHARE), Wave 1-7. Based on Release 7.0.0. Munich: SHARE-ERIC: 2019.

28 Sanchez-Niubo A, Egea-Cortés L, Olaya B, *et al.* Cohort Profile: The Ageing Trajectories of Health - Longitudinal Opportunities and Synergies (ATHLOS) project. *Int J Epidemiol* 2019;**48**:1052-1053I. doi:10.1093/ije/dyz077

29 World Health Organization. World report on ageing and health 2015. 2015.https://www.who.int/ageing/events/world-report-2015-launch/en/ (accessed 27 Jul 2020).

30 Berlin KS, Parra GR, Williams NA. An Introduction to Latent Variable Mixture Modeling (Part 2): Longitudinal Latent Class Growth Analysis and Growth Mixture Models. *J*

For peer review only - http://bmjopen.bmj.com/site/about/guidelines.xhtml

Pediatr Psychol 2014;**39**:188–203. doi:10.1093/jpepsy/jst085

31 Reischies F, Lobo A, Turrina C, *et al.* Development of the EURO–D scale – a European Union initiative to compare symptoms of depression in 14 European centres. *Br J Psychiatry* 2008;**174**:330–8. doi:10.1192/bjp.174.4.330

32 Copeland JRM, Beekman ATF, Dewey ME, *et al.* Depression in Europe. Geographical distribution among older people. *Br J Psychiatry* 1999;**174**:312–21. doi:10.1192/bjp.174.4.312

33 WHO. Country groupings. https://www.who.int/quantifying_ehimpacts/global/ebdcountgroup/en/ (7 February 2019, date last accessed)

34 United Nations Statistical Division (UNSD). Countries or areas/geographical regions. https://unstats.un.org/unsd/methodology/m49/ (7 February 2019, date last accessed)

35 Tein JY, Coxe S, Cham H. Statistical power to detect the correct number of classes in latent profile analysis. *Struct Equ Model* 2013;**20**:640–57. doi:10.1080/10705511.2013.824781

36 Nylund-Gibson K, Choi AY. Ten frequently asked questions about latent class analysis. *Transl Issues Psychol Sci* 2018;**4**:440–61. doi:10.1037/tps0000176

37 Aalen O. A Model for Nonparametric Regression Analysis of Counting Processes. Springer, New York, NY 1980. 1–25. doi:10.1007/978-1-4615-7397-5_1

38 Aalen OO. A linear regression model for the analysis of life times. *Stat Med* 1989;**8**:907–25. doi:10.1002/sim.4780080803

39 O. O. Aalen; O. Borgan; H. K. Gjessing. *Survival and event history analysis: a process point of view*. Springer. New York: 2008.

40 Xie X, Strickler HD, Xue X. Additive hazard regression models: An application to the natural history of human papillomavirus. Comput. Math. Methods Med. 2013;**2013**. doi:10.1155/2013/796270

41 Aalen OO, Scheike TH. Aalen's Additive Regression Model. In: *Encyclopedia of Biostatistics*. Chichester, UK: : John Wiley & Sons, Ltd 2005. doi:10.1002/0470011815.b2a11002

42 Team RC. R: A language and environment for statistical computing. 2020.https://www.r-project.org/

43 Lewis DR, Clegg LX, Johnson NJ. Lung disease mortality in the United States: the National Longitudinal Mortality Study. *Int J Tuberc Lung Dis* 2009;**13**:1008–14./pmc/articles/PMC2765862/?report=abstract (accessed 4 Feb 2021).

44 Manfreda J, Mao Y, Litven W. Morbidity and mortality from chronic obstructive pulmonary disease. *Am Rev Respir Dis* 1989;**140**:16–26. doi:10.1164/ajrccm/140.3_pt_2.s19

45 Ntritsos G, Franek J, Belbasis L, *et al.* Gender-specific estimates of COPD prevalence: a systematic review and meta-analysis. *Int J Chron Obstruct Pulmon Dis* 2018;**13**:1507–14. doi:10.2147/COPD.S146390

46 Hummer RA, Hernandez EM. The effect of educational attainment on adult mortality in the United States. *Popul Bull* 2013;**68**:1–

For peer review only - http://bmjopen.bmj.com/site/about/guidelines.xhtml

16.http://www.ncbi.nlm.nih.gov/pubmed/25995521 (accessed 9 Dec 2020).

47 OECD/European Union. Mortality from respiratory diseases. In: OECD Publishing, ed. *Health at a Glance: Europe 2018: State of Health in the EU Cycle*. Paris/European Union, Brussels: 2018. doi:https://doi.org/10.1787/health_glance_eur-2018-en

48 Lange P, Tøttenborg SS, Sorknæs AD, *et al.* Danish register of chronic obstructive pulmonary disease. *Clin Epidemiol* 2016;**8**:673–8. doi:10.2147/CLEP.S99489

49 Camozzato AL, Godinho C, Chaves MLF. Effect of successful aging on mortality in older individuals: the PALA study. *Dement e Neuropsychol* 2014;**8**:182–6. doi:10.1590/S1980-57642014DN82000015

50 O'Connell MDL, Marron MM, Boudreau RM, *et al.* Mortality in relation to changes in a healthy aging index: the Health, Aging, and Body Composition Study. *Journals Gerontol - Ser A Biol Sci Med Sci* 2019;**74**:726–32. doi:10.1093/gerona/gly114

51 Mannino DM. COPD: epidemiology, prevalence, morbidity and mortality, and disease heterogeneity. *Chest* 2002;**121**:121–6. doi:10.1378/chest.121.5_suppl.121S

52 Janson C, Marks G, Buist S, *et al.* The impact of COPD on health status: Findings from the BOLD study. *Eur Respir J* 2013;**42**:1472–83. doi:10.1183/09031936.00153712

53 López Varela MV, Montes de Oca M, Halbert R, *et al.* Comorbidities and Health Status in Individuals With and Without COPD in Five Latin American Cities: The PLATINO Study. *Arch Bronconeumol* 2013;**49**:468–74. doi:10.1016/j.arbr.2013.09.009

54 Jones PW, Quirk FH, Baveystock CM. The St George's Respiratory Questionnaire. *Respir Med* 1991;**85**:25–31. doi:10.1016/S0954-6111(06)80166-6

55 Celli BR, Cote CG, Marin JM, *et al.* The Body-Mass Index, Airflow Obstruction, Dyspnea, and Exercise Capacity Index in Chronic Obstructive Pulmonary Disease. *N Engl J Med* 2004;**350**:1005–12. doi:10.1056/nejmoa021322

56 Huntley AL, Johnson R, Purdy S, *et al.* Measures of multimorbidity and morbidity burden for use in primary care and comminuty settings: a systematic review and guide. *Ann Fam Med* 2012;**10**:134–41. doi:10.1370/afm.1363

57 Chapmann KR, Mannino DM, Soriano JB, *et al.* Epidemiology and costs of chronic obstructive pulmonary disease. Eur. Respir. J. 2006;**27**:188–207. doi:10.1183/09031936.06.00024505

58 Quaderi SA, Hurst JR. The unmet global burden of COPD. Glob. Heal. Epidemiol. Genomics. 2018;**3**:1–3. doi:10.1017/gheg.2018.1

For peer review only - http://bmjopen.bmj.com/site/about/guidelines.xhtml

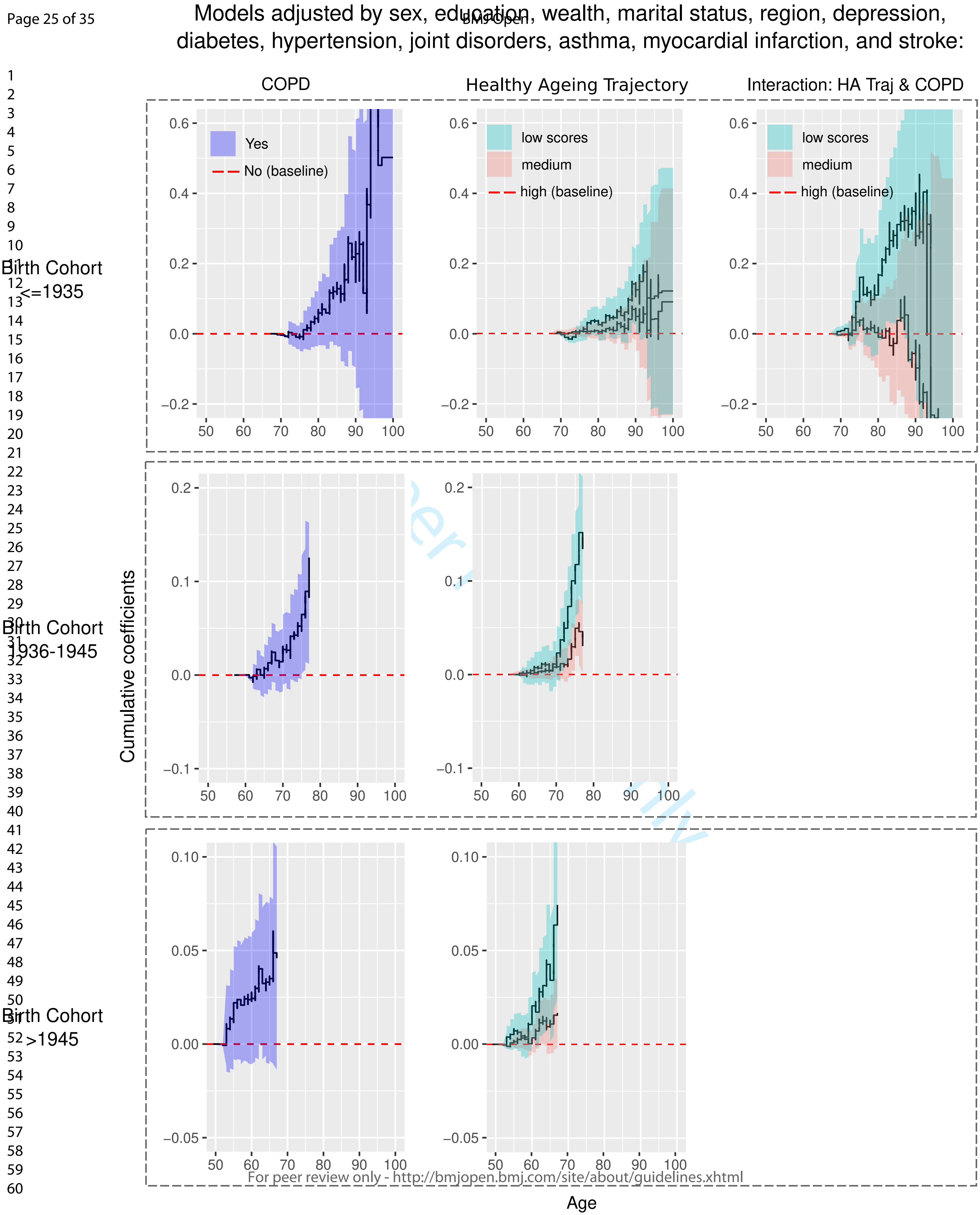
Models adjusted by sex, education, wealth, marital status, region, depression, diabetes, hypertension, joint disorders, asthma, myocardial infarction, and stroke:
COPD
Healthy Ageing Trajectory
Interaction: HA Traj & COPD
Yes
No (baseline)
low scores
medium
high (baseline)
Birth Cohort <=1935
Birth Cohort 1936-1945
Birth Cohort >1945
Cumulative coefficients
Age

Supplemental material

Supplementary Table 1. Harmonised items of the healthy ageing scale in the SHARE study

ATHLOS harmonized variables			SHARE
Memory	Self-reported ratings of memory at the time of the interview	Label	A4-B3 Memory self-rating
		Values	1 = None; 2 = Mild; 3 = Moderate; 4 = Severe; 5 = Extreme
		Harmonisation	1 = Absence; 2-5 = Presence
Immediate recall	Immediate recall of common nouns from a list	Label	ten words list learning first trial total
		Values	Continuous Variable 0-10
		Harmonisation	<=25% into Presence<br>>25% into Absence
Delayed recall	Test that assesses delayed recall using the common nouns from the list previously employed for measuring Immediate recall	Label	ten words list learning delayed recall total
		Values	Continuous Variable 0-10
		Harmonisation	<=25% into Presence<br>>25% into Absence
Verbal fluency†	Test that assesses verbal (semantic) fluency	Label	verbal fluency score
		Values	Continuous Variable 0-88
		Harmonisation	<=25% into Presence<br>>25% into Absence
Orientation in time	Difficulties for orientation in time, evaluated by a set of questions about the date and day of the week	Label	orientation to date, month, year and day of week
		Values	0-3 = bad; 4 = good
		Harmonisation	4 = Absence; 0-3 = Presence
Numeracy		Label	cf012_: Chance disease 10 perc. of 1000<br>cf013_: Half price [of a 300 Euro sofa]<br>cf014_: 6000 is two-thirds what is total price<br>cf015_: Amount in the savings account [on 2000 Euros after 2 years of 10% interest]
		Values	All: 1 = correct answer: 100; 2 = wrong answer: 10; 3 = wrong answer: 90; 4 = wrong answer: 900; 97 = wrong answer: Other answer keep 1 into 1; recode -1, 2, 3, 4, 5, 6 and 97 into 0
		Harmonisation	All 1 = Absence; Some 0 = Presence
Sleeping	Sleeping problems	Label	sleep (part of EURO-D)
		Values	0 = Not selected; 1 = Selected
		Harmonisation	0 = Absence; 1 = Presence
Pain	It measures if the participant experiences some degree of pain or if the participant does not present any pain at all	Label	bothered by: pain in back, knees, hips or other joint
		Values	0 = Not selected; 1 = Selected
		Harmonisation	0 = Absence; 1 = Presence
Energy	Self-reported high level of energy experienced at the time of the interview	Label	fatigue
		Values	1 = Yes; 5 = No
		Harmonisation	5 = Absence; 1 = Presence
		Label	bothered by: incontinence

For peer review only

Urine incontinence	It measures if the participant has experienced loss of urine (or has used any special device for urine leakage)	Values	0 = Not selected; 1 = Selected
		Harmonisation	0 = Absence; 1 = Presence
Near vision	Difficulties for near vision	Label	eyesight reading
		Values	1 = excellent; 2 = very good; 3 = good; 4 = fair; 5 = poor
		Harmonisation	1-3 = Absence; 4-5 = Presence
Far vision	Difficulties for far vision	Label	eyesight distance
		Values	1 = excellent; 2 = very good; 3 = good; 4 = fair; 5 = poor
		Harmonisation	1-3 = Absence; 4-5 = Presence
Eyesight	Difficulties in eye sight using glasses or corrective lens as usual	Label	Is your eyesight (using glasses or contact lens if you use them)
		Values	1 = excellent; 2 = very good; 3 = good; 4 = fair; 5 = poor; 6 = registered or legally blind
		Harmonisation	1-3 = Absence; 4-6 = Presence
Hearing in general	It measures if the participant experiences some difficulty for hearing (i.e., hearing someone talking on the other side of the room in a normal voice) or not, using a hearing aid as usual	Label	hearing
		Values	1 = excellent; 2 = very good; 3 = good; 4 = fair; 5 = poor
		Harmonisation	1-3 = Absence; 4-5 = Presence
Hearing in a conversation	It measures if the participant experiences some difficulty (including total disability) for following a conversation (i.e., if there is a background noise, or several people talking) or not, using a hearing aid as usual	Label	hearing with one person
		Values	1 = Yes; 5 = No
		Harmonisation	1 = Absence; 0= Presence
Stooping, kneeling or crouching	Difficulty for stooping, kneeling or crouching	Label	difficulties: stooping, kneeling, crouching
		Values	0 = Not selected; 1 = Selected
		Harmonisation	0 = Absence; 1 = Presence
Lifting or carrying weights	Difficulty for lifting or carrying weights	Label	difficulties: lifting or carrying weights over 5 kilos
		Values	0 = Not selected; 1 = Selected
		Harmonisation	0 = Absence; 1 = Presence
Climbing stairs	Difficulty for climbing stairs	Label	difficulties: climbing one flight of stairs
		Values	0 = Not selected; 1 = Selected
		Harmonisation	0 = Absence; 1 = Presence
Getting up	Difficulty for getting up from sitting down	Label	difficulties: getting up from chair
		Values	0 = Not selected; 1 = Selected
		Harmonisation	0 = Absence; 1 = Presence
Walking	Difficulty for walking by yourself and without using any special equipment	Label	difficulties: walking 100 metres
		Values	0 = Not selected; 1 = Selected
		Harmonisation	0 = Absence; 1 = Presence
Pulling or pushing	Difficulty for pulling or pushing large objects	Label	difficulties: pulling or pushing large objects
		Values	0 = Not selected; 1 = Selected
		Harmonisation	0 = Absence; 1 = Presence
Sitting	Difficulty for sitting for long periods	Label	difficulties: sitting two hours
		Values	0 = Not selected; 1 = Selected

For peer review only

		Harmonisation	0 = Absence; 1 = Presence
Reaching or extending arms	Difficulty for reaching / extending arms	Label	difficulties: reaching or extending arms above shoulder
		Values	0 = Not selected; 1 = Selected
		Harmonisation	0 = Absence; 1 = Presence
Walking speed	It is measured assessing the time that is taken to walk a distance	Label	walking speed
		Values	Continuous
		Harmonisation	<=25% into Presence<br>>25% into Absence
Dizziness	Dizziness problems when walking on a level surface	Label	bothered by: dizziness, faints or blackouts
		Values	0 = Not selected; 1 = Selected
		Harmonisation	0 = Absence; 1 = Presence
Picking up	Difficulty for picking up things with fingers, e.g. picking up a coin	Label	difficulties: picking up a small coin from a table
		Values	0 = Not selected; 1 = Selected
		Harmonisation	0 = Absence; 1 = Presence
Getting in or out of bed	Difficulty for getting in or out of bed	Label	difficulties: getting in or out of bed
		Values	0 = Not selected; 1 = Selected
		Harmonisation	0 = Absence; 1 = Presence
Bathing or showering	Difficulties for bathing or showering	Label	difficulties: bathing or showering
		Values	0 = Not selected; 1 = Selected
		Harmonisation	0 = Absence; 1 = Presence
Getting dressed	Difficulty for getting dressed	Label	difficulties: dressing, including shoes and socks
		Values	0 = Not selected; 1 = Selected
		Harmonisation	0 = Absence; 1 = Presence
Moving around the home	Difficulty for moving around the home	Label	difficulties: walking across a room
		Values	0 = Not selected; 1 = Selected
		Harmonisation	0 = Absence; 1 = Presence
Toilet	Difficulties for using the toilet	Label	difficulties: using the toilet, incl getting up or down
		Values	0 = Not selected; 1 = Selected
		Harmonisation	0 = Absence; 1 = Presence
Eating	Difficulties for eating	Label	difficulties: eating, cutting up food
		Values	0 = Not selected; 1 = Selected
		Harmonisation	0 = Absence; 1 = Presence
Housework	Difficulties for doing housework	Label	difficulties: doing work around the house or garden
		Values	0 = Not selected; 1 = Selected
		Harmonisation	0 = Absence; 1 = Presence
Shopping	Difficulties for shopping groceries	Label	difficulties: shopping for groceries
		Values	0 = Not selected; 1 = Selected
		Harmonisation	0 = Absence; 1 = Presence
Meals	Difficulties in preparing meals	Label	difficulties: preparing a hot meal
		Values	0 = Not selected; 1 = Selected
		Harmonisation	0 = Absence; 1 = Presence
Map	Difficulties for using a map	Label	difficulties: using a map in a strange place
		Values	0 = Not selected; 1 = Selected
		Harmonisation	0 = Absence; 1 = Presence
Money	Difficulties for managing money, bills, or expenses	Label	difficulties: managing money
		Values	0 = Not selected; 1 = Selected
		Harmonisation	0 = Absence; 1 = Presence
Medications	Difficulties for taking medications	Label	difficulties: taking medications

For peer review only

		Values	0 = Not selected; 1 = Selected
		Harmonisation	0 = Absence; 1 = Presence
Telephone	Difficulties for using telephone	Label	difficulties: telephone calls
		Values	0 = Not selected; 1 = Selected
		Harmonisation	0 = Absence; 1 = Presence

For peer review only

Supplementary Table 2. Results from the latent class growth analysis models for the three birth cohorts

No. of classes	Loglik	npm	BIC	% class 1	% class 2	% class 3	% class 4	% class 5
**≤1935**								
1	-83923.67	3	167875.0	100.00				
2	-80924.65	6	161904.6	51.39	48.60			
**3**	**-79920.76**	**9**	**159924.4**	**20.21**	**31.38**	**48.39**		
4	-79545.29	12	159201.1	7.37	32.59	23.22	36.80	
5	-79429.36	15	158996.8	31.64	23.08	1.31	7.19	36.76
**1936 – 1945**								
**No. of classes**	**Loglik**	**npm**	**BIC**	**% class 1**	**% class 2**	**% class 3**	**% class 4**	**% class 5**
1	-86218.80	3	172465.1	100.00				
2	-82811.31	6	165677.6	66.84	33.15			
**3**	**-81877.93**	**9**	**163838.3**	**49.72**	**12.44**	**37.83**		
4	-81611.24	12	163332.3	43.55	35.79	2.38	18.26	
5	-81540.03	15	163217.4	33.21	12.13	24.67	28.00	1.97
**>1945**								
**No. of classes**	**Loglik**	**npm**	**BIC**	**% class 1**	**% class 2**	**% class 3**	**% class 4**	**% class 5**
1	-89325.74	3	178679.1	100.00				
2	-85721.95	6	171499.2	23.37	76.62			
**3**	**-84856.16**	**9**	**169795.3**	**8.75**	**30.81**	**60.43**		
4	-84608.23	12	169327.1	30.97	53.19	13.34	2.47	
5	-84607.84	15	169354.0	2.39	13.17	32.36	52.02	0.02

Supplementary Figure 1. Trajectories of healthy ageing among birth cohorts

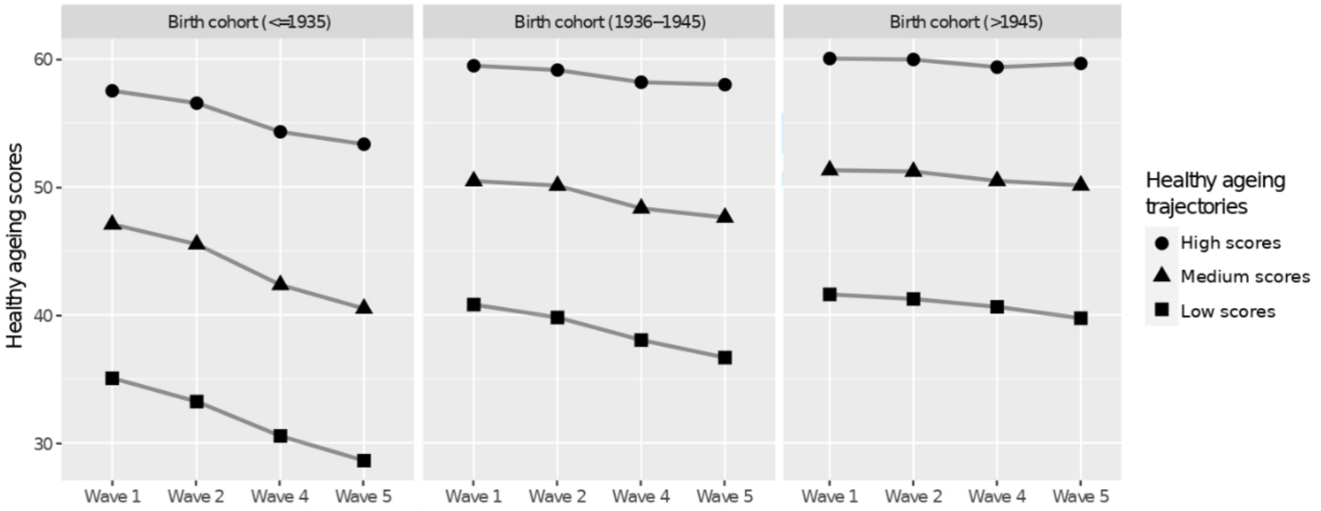

For peer review only - http://bmjopen.bmj.com/site/about/guidelines.xhtml

Supplementary Figure 2. Estimates of cumulative excess of risk of COPD and healthy ageing trajectories, separately by using the Aalen's additive regression

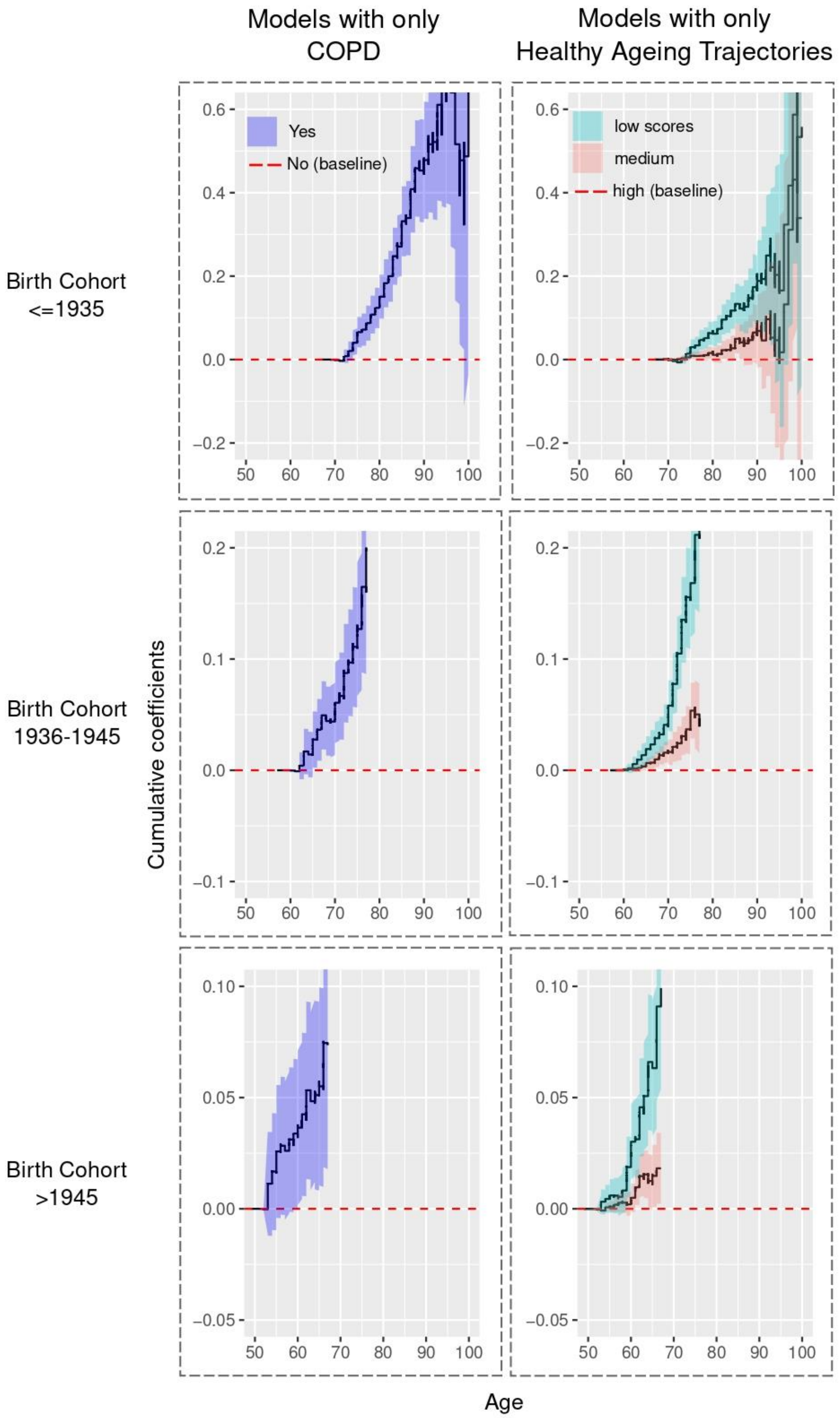

Supplementary Figure 3.

Figure 3.1. Estimates of cumulative excess risk of covariates from the Aalen's additive regression model by >1945 birth cohort sub-sample.

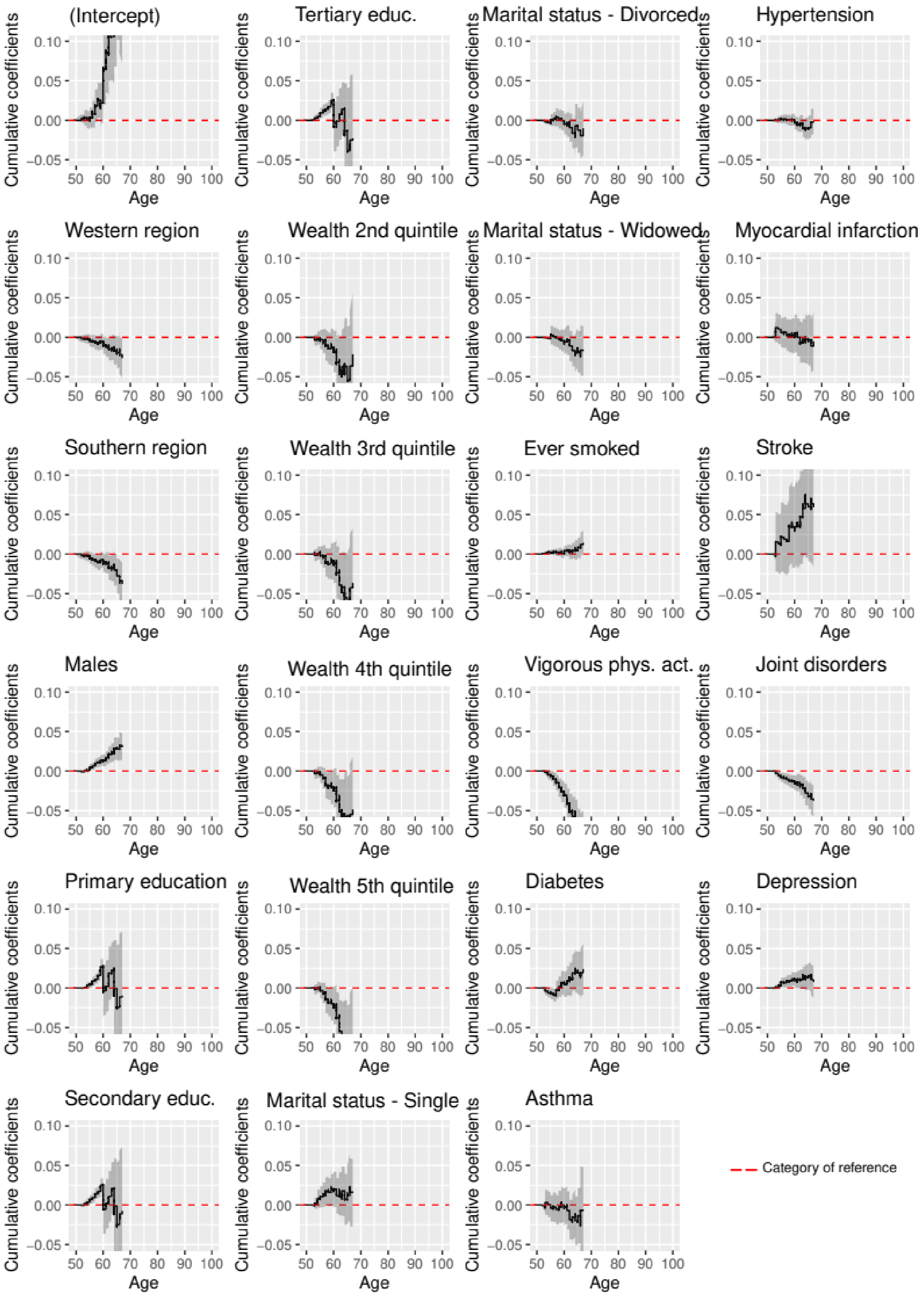

Figure 3.2. Estimates of cumulative excess risk of covariates from the Aalen's additive regression model by 1936-1945 birth cohort sub-sample.

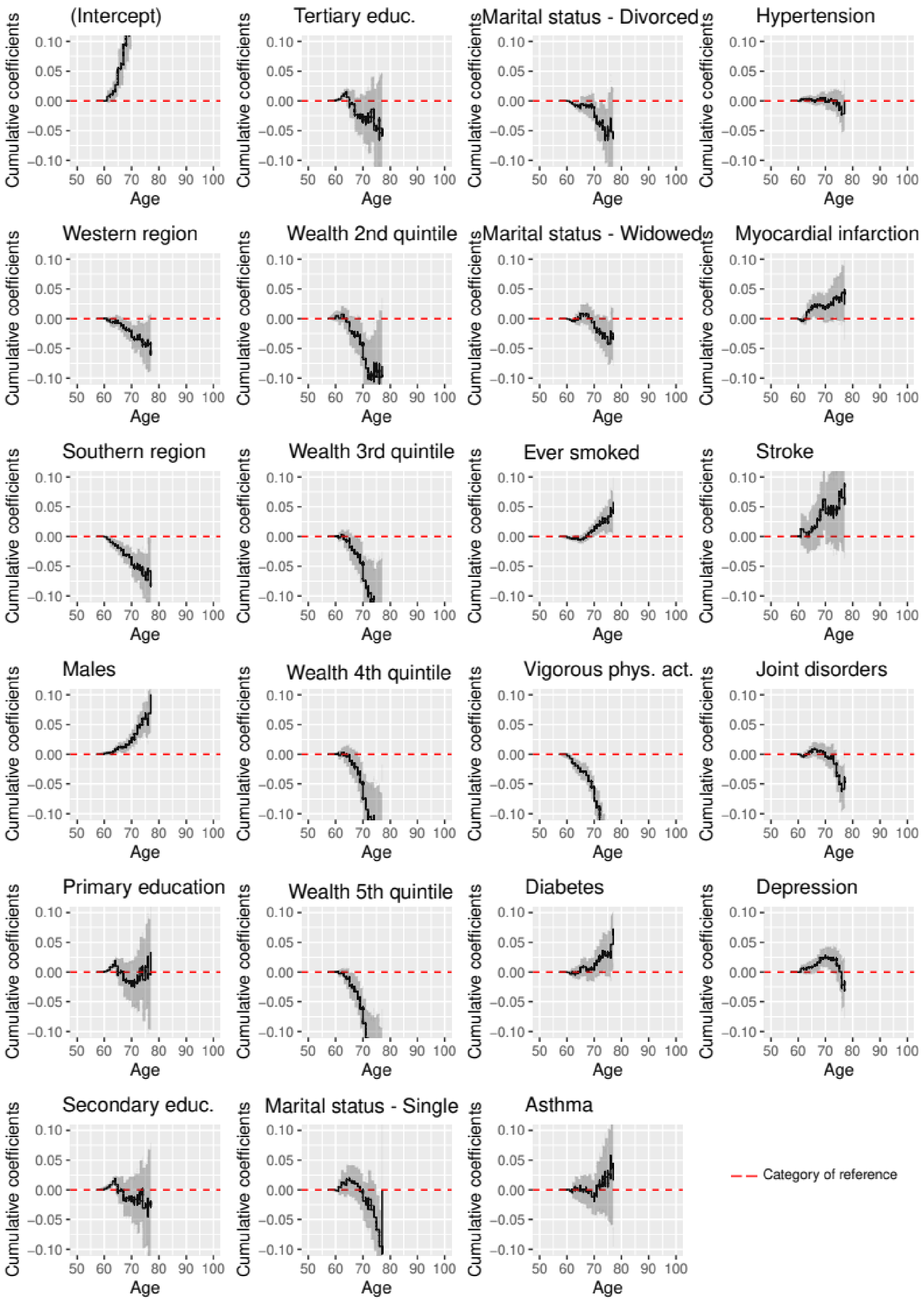

Figure 3.3. Estimates of cumulative excess risk of covariates from the Aalen's additive regression model by ≤1935 birth cohort sub-sample.

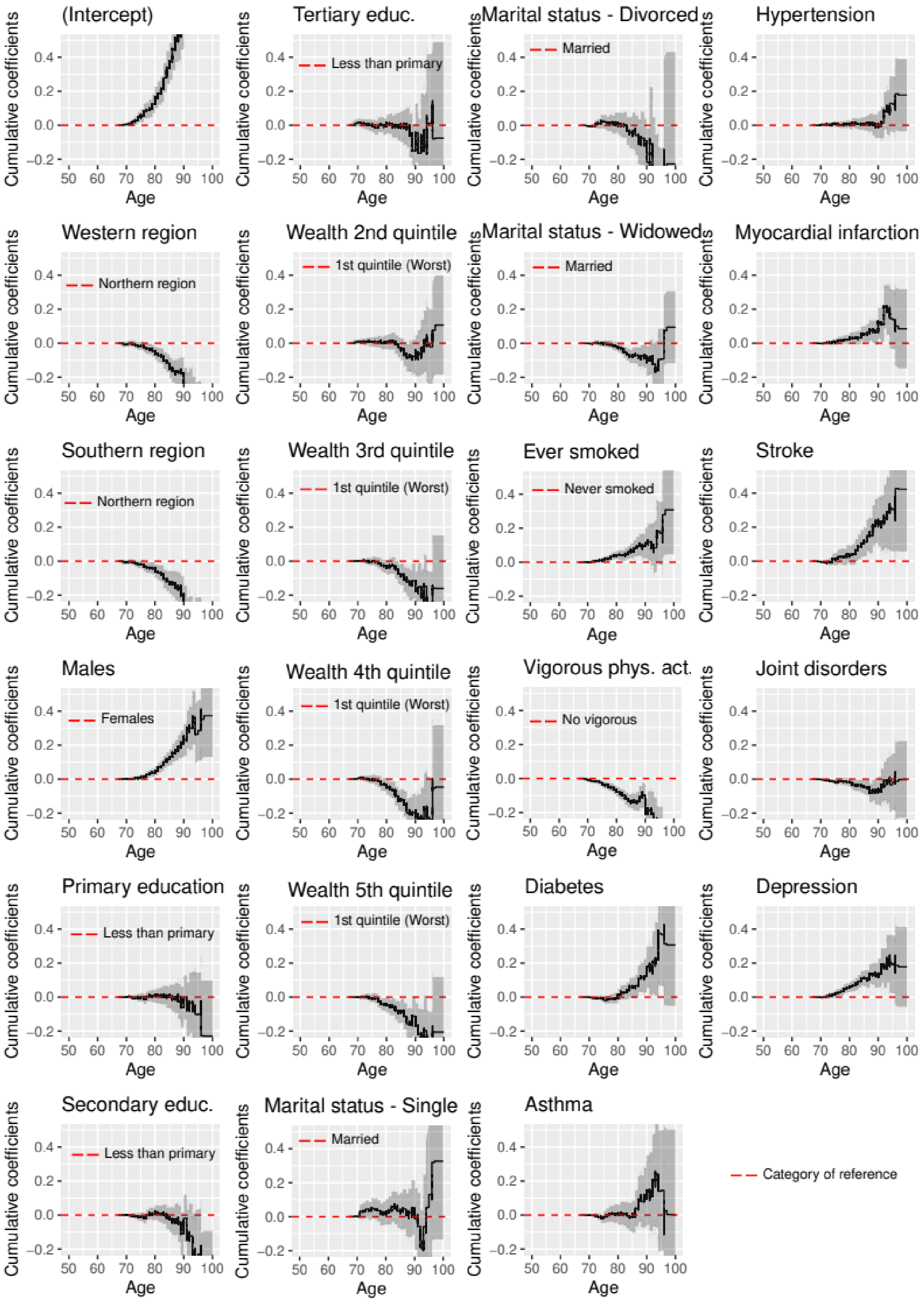

STROBE Statement—Checklist of items that should be included in reports of ***cohort studies***

	Item No	Recommendation	Page No
**Title and abstract**	1	(*a*) Indicate the study's design with a commonly used term in the title or the abstract<br>(*b*) Provide in the abstract an informative and balanced summary of what was done and what was found	1-2
**Introduction**			
Background/rationale	2	Explain the scientific background and rationale for the investigation being reported	3-6
Objectives	3	State specific objectives, including any prespecified hypotheses	5-6
**Methods**			
Study design	4	Present key elements of study design early in the paper	6
Setting	5	Describe the setting, locations, and relevant dates, including periods of recruitment, exposure, follow-up, and data collection	6
Participants	6	(*a*) Give the eligibility criteria, and the sources and methods of selection of participants. Describe methods of follow-up<br>(*b*) For matched studies, give matching criteria and number of exposed and unexposed	6-7
Variables	7	Clearly define all outcomes, exposures, predictors, potential confounders, and effect modifiers. Give diagnostic criteria, if applicable	7-9
Data sources/ measurement	8*	For each variable of interest, give sources of data and details of methods of assessment (measurement). Describe comparability of assessment methods if there is more than one group	7-9
Bias	9	Describe any efforts to address potential sources of bias	7-10
Study size	10	Explain how the study size was arrived at	6-7
Quantitative variables	11	Explain how quantitative variables were handled in the analyses. If applicable, describe which groupings were chosen and why	9-10
Statistical methods	12	(*a*) Describe all statistical methods, including those used to control for confounding<br>(*b*) Describe any methods used to examine subgroups and interactions<br>(*c*) Explain how missing data were addressed<br>(*d*) If applicable, explain how loss to follow-up was addressed<br>(*e*) Describe any sensitivity analyses	9-10
**Results**			
Participants	13*	(a) Report numbers of individuals at each stage of study—eg numbers potentially eligible, examined for eligibility, confirmed eligible, included in the study, completing follow-up, and analysed<br>(b) Give reasons for non-participation at each stage<br>(c) Consider use of a flow diagram	6-7
Descriptive data	14*	(a) Give characteristics of study participants (eg demographic, clinical, social) and information on exposures and potential confounders<br>(b) Indicate number of participants with missing data for each variable of interest<br>(c) Summarise follow-up time (eg, average and total amount)	10-11
Outcome data	15*	Report numbers of outcome events or summary measures over time	10-13

For peer review only - http://bmjopen.bmj.com/site/about/guidelines.xhtml

Main results	16	(*a*) Give unadjusted estimates and, if applicable, confounder-adjusted estimates and their precision (eg, 95% confidence interval). Make clear which confounders were adjusted for and why they were included<br>(*b*) Report category boundaries when continuous variables were categorized<br>(*c*) If relevant, consider translating estimates of relative risk into absolute risk for a meaningful time period	10-13
Other analyses	17	Report other analyses done—eg analyses of subgroups and interactions, and sensitivity analyses	Supplemental material
**Discussion**			
Key results	18	Summarise key results with reference to study objectives	17-18
Limitations	19	Discuss limitations of the study, taking into account sources of potential bias or imprecision. Discuss both direction and magnitude of any potential bias	16-17
Interpretation	20	Give a cautious overall interpretation of results considering objectives, limitations, multiplicity of analyses, results from similar studies, and other relevant evidence	13-18
Generalisability	21	Discuss the generalisability (external validity) of the study results	17-18
**Other information**			
Funding	22	Give the source of funding and the role of the funders for the present study and, if applicable, for the original study on which the present article is based	18

*Give information separately for exposed and unexposed groups.

Note: An Explanation and Elaboration article discusses each checklist item and gives methodological background and published examples of transparent reporting. The STROBE checklist is best used in conjunction with this article (freely available on the Web sites of PLoS Medicine at http://www.plosmedicine.org/, Annals of Internal Medicine at http://www.annals.org/, and Epidemiology at http://www.epidem.com/). Information on the STROBE Initiative is available at http://www.strobe-statement.org.

For peer review only

BMJ Open

Risk of all-cause mortality associated with chronic obstructive pulmonary disease and the role of healthy ageing trajectories: A population-based study of middle-aged and older adults.

Journal:	*BMJ Open*
Manuscript ID	bmjopen-2021-050947.R1
Article Type:	Original research
Date Submitted by the Author:	09-Jun-2021
Complete List of Authors:	Bayes-Marin, Ivet; Parc Sanitari Sant Joan de Déu, Research, Innovation and Teaching Unit; Universitat de Barcelona Facultat de Medicina<br>Sanchez-Niubo, Albert ; Parc Sanitari Sant Joan de Deu, ; Instituto de Salud Carlos III, Centro de Investigación Biomédica en Red de Salud Mental (CIBERSAM)<br>Fernández, Daniel; Polytechnic University of Catalonia, Serra Húnter fellow, Department of Statistics and Operations Research<br>Haro, Josep; Parc Sanitari Sant Joan de Deu, Research, Innovation and Teaching Unit; Centro de Investigación Biomédica en Red de Salud Mental<br>Olaya, Beatriz; Parc Sanitari Sant Joan de Deu, Research, Innovation and Teaching Unit; Centro de Investigación Biomédica en Red de Salud Mental
**Primary Subject Heading**:	Epidemiology
Secondary Subject Heading:	Public health, Respiratory medicine
Keywords:	Chronic airways disease < THORACIC MEDICINE, EPIDEMIOLOGY, PUBLIC HEALTH

SCHOLARONE™
Manuscripts

I, the Submitting Author has the right to grant and does grant on behalf of all authors of the Work (as defined in the below author licence), an exclusive licence and/or a non-exclusive licence for contributions from authors who are: i) UK Crown employees; ii) where BMJ has agreed a CC-BY licence shall apply, and/or iii) in accordance with the terms applicable for US Federal Government officers or employees acting as part of their official duties; on a worldwide, perpetual, irrevocable, royalty-free basis to BMJ Publishing Group Ltd ("BMJ") its licensees and where the relevant Journal is co-owned by BMJ to the co-owners of the Journal, to publish the Work in this journal and any other BMJ products and to exploit all rights, as set out in our licence.

The Submitting Author accepts and understands that any supply made under these terms is made by BMJ to the Submitting Author unless you are acting as an employee on behalf of your employer or a postgraduate student of an affiliated institution which is paying any applicable article publishing charge ("APC") for Open Access articles. Where the Submitting Author wishes to make the Work available on an Open Access basis (and intends to pay the relevant APC), the terms of reuse of such Open Access shall be governed by a Creative Commons licence – details of these licences and which Creative Commons licence will apply to this Work are set out in our licence referred to above.

Other than as permitted in any relevant BMJ Author's Self Archiving Policies, I confirm this Work has not been accepted for publication elsewhere, is not being considered for publication elsewhere and does not duplicate material already published. I confirm all authors consent to publication of this Work and authorise the granting of this licence.

For peer review only

Risk of all-cause mortality associated with chronic obstructive pulmonary disease and the role of healthy ageing trajectories: A population-based study of middle-aged and older adults.

Ivet Bayes-Marin [a,b,c], Albert Sanchez-Niubo [a,b], Daniel Fernández[d], Josep Maria Haro [a,b,c], and Beatriz Olaya [a,b]

[a]Research, Innovation and Teaching Unit, Parc Sanitari Sant Joan de Déu, Sant Boi de Llobregat, 08830 Barcelona, Spain.

[b]Centro de Investigación Biomédica en Red de Salud Mental (CIBERSAM), Instituto de Salud Carlos III, Madrid 28029, Spain.

[c]Department of Medicine, Universitat de Barcelona, Barcelona 08036, Spain.

[d]Serra Húnter fellow, Department of Statistics and Operations Research, Polytechnic University of Catalonia-BarcelonaTech, 08028 Barcelona, Spain.

***Corresponding author:**

Albert Sanchez-Niubo. Research, Innovation and Teaching Unit, Parc Sanitari Sant Joan de Déu, Dr Antoni Pujadas 42, Sant Boi de Llobregat, Barcelona 08830, Spain. E-mail: albert.sanchez@pssjd.org

Word count: 5840

Abstract

Objectives: The aims were to study the risk of all-cause mortality associated with chronic obstructive pulmonary disease (COPD) and healthy ageing trajectories (HAT) in three birth cohorts and to determine the moderating role of HAT in the association between COPD and all-cause mortality. **Design:** prospective cohort study. **Setting:** Data from waves 1 to 5 of The

For peer review only - http://bmjopen.bmj.com/site/about/guidelines.xhtml

Survey of Health, Ageing and Retirement in Europe (SHARE). **Participants:** The total sample was 28,857 community-dwelling individuals aged 50+ years. **Main outcome:** All-cause mortality associated with COPD and HAT adjusting for covariates. We performed Aalen additive hazards models to explore these associations. Interactions between COPD and HAT were also explored. Analyses were conducted separately in three birth cohorts (>1945, 1936-1945, and ≤1935). Latent class growth analysis was used to classify participants into HAT. **Results:** Three parallel HAT were found in the three birth cohorts ("low", "medium", and "high" healthy ageing). Participants with COPD had an increased mortality risk, but this effect was no longer significant after adjusting for covariates. The "low" HAT was associated with increased mortality risk in the three sub-samples, although this effect was lower after adjustment. The interaction between COPD and HAT was significant only in the ≤1935 birth cohort, indicating that those with COPD and a "low" trajectory had a greater risk of mortality. **Conclusions:** The healthy ageing scale may be a suitable tool to identify patients at higher risk to mitigate disease burden and improve patients' quality of life.

Keywords: chronic obstructive pulmonary disease (COPD), mortality, healthy ageing, Europe, population-based study

Strengths and limitations of this study

- The analyses were performed in different birth cohorts (>1945, 1936-1945, and ≤1935) to assess differences in mortality risks related to societal changes, such as lifestyle behaviours and occupation trends.
- We used a novel measurement scale of healthy ageing including intrinsic capacity and functional ability variables.
- The calculation of Aalen additive hazards models rather than Cox models allowed the inclusion of time-variant variables in the analyses.

For peer review only - http://bmjopen.bmj.com/site/about/guidelines.xhtml

- Due to the high percentage of missingness in the age of the diagnosis of the diseases, we selected the age of the earliest diagnosis of each disease within the five waves.
- For the survival analysis we used the age of the participants instead of the years of the interview for better interpretation. However, this introduces a problem of left truncation since the age range observed for each participant is different, although we took this into account in the additive regression model.

Introduction

Chronic obstructive pulmonary disease (COPD) is a major cause of morbidity and mortality worldwide(1,2). COPD is expected to become the third leading cause of death by 2030(3). The growing burden of COPD is a consequence of population ageing and the continued use of tobacco, which is considered its main risk factor(4). Moreover, air pollution has been associated with acute exacerbations of COPD, increased respiratory morbidity and mortality(5).

COPD is characterised by a progressive airflow limitation associated with an abnormal inflammatory response of the lungs to noxious particles or gases(6). With a worsening of the disease, there can be an increase of functional ability limitations in the activities of daily living (ADL), and in the instrumental activities of daily living (IADL), limiting exercise performance and self-care(7–9). COPD has increasingly been recognised as a multi-component disease, associated with a wide range of physical diseases and psychological disorders(10). Non-communicable diseases (NCDs) such as hypertension, cardiovascular diseases, diabetes, cancer, and depression commonly co-exist in COPD patients, worsening its progression(1,2,11). Furthermore, cognitive impairment is common among COPD patients, suggesting that impaired performance in neuropsychological tests might be a predictor of early mortality for people diagnosed with COPD(10).

For peer review only - http://bmjopen.bmj.com/site/about/guidelines.xhtml

Despite being a growing public health concern, there is a lack of epidemiological data about the prevalence and distribution of COPD(6,12). The paucity of information on COPD prevalence and incidence is partly due to differences in the methods used for its diagnosis and classification, often being underestimated(6). These differences in the assessment methods and definitions have also hampered the comparison of COPD prevalence and impact across countries. Both The Burden of Lung Disease (BOLD) project(4) and the Latin American Project for the Investigation of Obstructive Lung Disease (PLATINO)(13) were developed to map the COPD prevalence using the same methodology in different countries. Those studies were performed in China and Turkey, and five Latin American countries, respectively. Nevertheless, there is a lack of integrated and updated estimates of COPD prevalence, similar to BOLD and PLATINO initiatives, and information regarding patient's quality of life impact and associated mortality in Europe(2,14). Additionally, available studies suggest large differences across European countries regarding prevalence rates of COPD and the associated death rates(15,16). In a systematic review, COPD prevalence ranged from 3% in Finnish women to 57% in Italian men and women(15). Some differences have also been found in COPD-related mortality across European countries and between men and women(16). Overall, regarding European countries, COPD-related mortality rates appeared to decline in men in most countries from 1995 to 2017, whereas mortality rates due to COPD increased in women from +2% per year in Austria to +4.2% or +4.8% per year in the Czech Republic and Hungary, respectively(16).

In that sense, an integrated longitudinal dataset that considers different European countries could be particularly useful in studying the risk of mortality associated with COPD in different European countries. In particular, using a cross-national panel database may prevent possible heterogeneities arising from differences in survey methodologies, diagnostic criteria, and population structure(17). The study of the mortality risk in COPD patients, and its association with several variables related to health and functioning would allow identifying those

For peer review only

For peer review only - http://bmjopen.bmj.com/site/about/guidelines.xhtml

vulnerable sectors of the population and creating of preventive measures and interventions in diverse healthcare systems.

Previous studies focused on the association between exercise capacity and mortality among COPD patients, which has been considered one of the best predictors of mortality(18–20). Measures of exercise capacity include indicators such as body mass index, airflow obstruction, dyspnea, handgrip strength, and the sit-to-stand test(21). Nevertheless, these indicators of exercise capacity are just measures of intrinsic capacity that do not capture the individual's functional ability over the life course. In that sense, the functional ability results from the interaction of the individuals' intrinsic capacity, including physical and mental capacities, and their environment, as access to medications, personal and assistive support, or physical barriers(22). Therefore, a measure assessing both intrinsic capacity and functional ability may be a better way to capture a person's healthy ageing.

Several authors advocate for using composite measures as the International Classification of Functioning, Disability, and Health to assess COPD patients' complexity, including also functional capacity and functional performance(23). Related to this, the Ageing Trajectories of Health: Longitudinal Opportunities and Synergies (ATHLOS) project(24) developed a healthy ageing scale(22) using 16 international cohort studies to determine the intrinsic capacity and functional ability of the participants allowing comparisons across countries. The healthy ageing scale comprises several domains such as vitality, sensory skills, locomotion/mobility, cognition, ADL, and IADL. Thus, this measure includes measures of exercise capacity and functionality that could be affected by the course of COPD disease and impact on the patients' quality of life.

The aims of the present paper are: 1) to study the risk of all-cause mortality associated with COPD and healthy ageing trajectories (HAT) in three population-based cohorts of middle-aged and older adults; 2) to determine the moderating role of HAT in the association between COPD

For peer review only - http://bmjopen.bmj.com/site/about/guidelines.xhtml

and all-cause mortality. We speculated that a HAT characterised by low levels of healthy ageing would be significantly associated with an increased risk of mortality in people with COPD. In contrast, individuals with higher levels of healthy ageing and COPD would have a lower risk of mortality.

Methods

Study design and Data Collection

The present study used data from five waves of The Survey of Health, Ageing and Retirement in Europe (SHARE)(25). SHARE is a multidisciplinary, cross-national panel database that contains a broad range of information on health, socioeconomic status, and social networks of European citizens aged 50 and older. The first wave took place in 2004-2005, constituted by more than 22,000 persons born in 1954 and earlier, and the following waves were conducted approximately every two years. The interviewers used computer-assisted personal interviewing (CAPI) to collect most of the data in all waves. Additionally, in waves 1, 2, and 4, self-administered questionnaires were handed out after the CAPI completion. If a respondent passed away during the study, then an end-of-life interview was conducted with a proxy.

The overall individual response rate at baseline was 60.1%, and the wave-to-wave retention rate of participants from wave 1 was higher than 55% in all the countries(26). All participants gave written consent. Ethical approvals for waves from 1 to 3 were granted by the Ethics Committee of the University of Mannheim(25). For waves 4 and 5, the SHARE projects were reviewed and approved by the Ethics Council of the Max-Planck Society(27). Further details concerning the study design of SHARE can be found elsewhere(25).

The following countries were included in the present analysis: Denmark, Sweden, Greece, Italy, Spain, Israel, Austria, Belgium, France, Germany, Netherlands, and Switzerland. We excluded participants incorporated in the subsequent waves due to the sample's refreshments

For peer review only - http://bmjopen.bmj.com/site/about/guidelines.xhtml

(*n*=30,816). The analyses focused on people aged 50 years and older who completed a non-proxy interview at baseline, resulting in an analytical sample of 28,857 respondents.

Patient and public involvement

No patient involved.

Measurements

- *All-cause mortality*

The death of a participant was confirmed by interviewing a proxy-respondent since information on the deceased was not linked to national death registries(26,28). If confirmed, the date of death was obtained from end-of-life interviews with a proxy respondent(26,28). Participants were characterised as survivors or censored if they were alive at the end of the study period, and dead if they died during the study period.

Survival time was calculated in years and as follows: 1) from baseline to the reported date of death or the final assessment date for those participants who were alive at the end of 2013; or 2) in the case that a participant reported being diagnosed with COPD at baseline, survival time was calculated from baseline. Besides, for the set of patients who reported a new diagnosis of COPD during the follow-up period, we considered the first time of the observation as the age at which they were newly diagnosed.

- *Chronic obstructive pulmonary disease (COPD)*

Participants reported whether a doctor ever informed them that they had “COPD such as chronic bronchitis or emphysema”. In the present study, we considered the first age in which a participant reported having been diagnosed with COPD instead of considering the presence/absence of COPD at baseline because the participant might be diagnosed in the subsequent waves. Therefore, COPD diagnosis was treated as a time-variant variable.

- *Healthy ageing scale*

For peer review only - http://bmjopen.bmj.com/site/about/guidelines.xhtml

We used an international scale of healthy ageing measurement developed by the ATHLOS consortium(22,29). This scale used items about intrinsic capacity and functional ability based on the World Mental Health's (WHO) concept of healthy ageing(30). The healthy ageing scale covers different domains, such as vitality, sensory skills, locomotion/mobility, cognition, ADL, and IADL. Thirty-nine study-specific variables were harmonised into dichotomous items indicating the presence or absence of difficulties (see Supplementary Table 1). Final scores were estimated for all individuals and converted to *T*-scores with a mean of 50 and a standard deviation of 10. We applied latent class growth analysis (LCGA)(31) to identify longitudinal trajectories according to the healthy ageing scale score across the waves and classify the participants into those trajectories.

- *Covariates*

Demographic variables included sex (male/female), age (in years), level of education (less than primary, primary, secondary, and tertiary), marital status (single, married or currently cohabiting, separated or divorced, and widowed), and quintiles of household wealth (first quintile indicating lowest level).

Lifestyles and health behaviours included ever smoked and practice of vigorous physical activity during the last two weeks and were coded as *yes* or *no*. The following self-reported diagnoses of NCDs different from COPD were included: diabetes, hypertension, joint disorders (arthritis, rheumatism, or osteoarthritis), asthma, myocardial infarction, and stroke. Similar to COPD, we selected the age of the earliest diagnosis of each NCD across the five waves, considering them as time-variant variables.

Depression was assessed with the EURO-D 12-item scale, which was developed and validated for the EURODEP studies to measure depressive symptoms across European countries, accounting for regional differences(32,33). The EURO-D score ranges from 0 to 12, with higher

For peer review only - http://bmjopen.bmj.com/site/about/guidelines.xhtml

scores meaning higher levels of depression, being four or greater than the proposed cut-off score that has been selected to create a dichotomous depression variable (yes/no)(32).

Finally, we grouped the countries into three European regions according to the World Health Organization (WHO) and the United Nations Statistical Division (UNSD) regional classification(34,35). Thus, Northern Europe was constituted by Denmark and Sweden; Western Europe included Austria, Belgium, France, Germany, Israel, the Netherlands; and Switzerland, and Southern Europe included Spain, Italy, and Greece.

Statistical Analyses

We divided the sample into three groups according to the participants' birth year and kept proportional sample sizes. The first group (*n*=9,866) was composed of those participants who were born after 1945 (the youngest participants: aged 50+), the second group (*n*=9,254) comprised participants born between 1936 and 1945 (ages from 58 to 70 years old), and the third one (*n*=9,739) encompassed individuals who were born in 1935 or earlier (the oldest participants: from 69 to 104 years old). Analyses were independently conducted in these three birth cohorts.

Latent class growth analysis (LCGA) was used to classify individuals into trajectories based on their score on the healthy ageing scale(31). The number of trajectories was determined by analysing group models from 1 to 5 trajectories. According to the Bayesian information criterion, the optimal model was selected. The lowest value indicates the better fit(36,37) and the sample size of the trajectory group. In addition, a sample size lower than 5% was considered insufficient to identify classes(37).

To analyse the associations between COPD and time to death, we conducted an Aalen additive hazards modelling approach, avoiding the assumption of proportionality of the Cox regression hazards (38,39). These models can provide a better picture of how the effects of covariates

For peer review only - http://bmjopen.bmj.com/site/about/guidelines.xhtml

develop over time without assuming the proportional risk hypothesis as in the Cox regression models(40). Parameters of these models are arbitrary cumulative regression functions that represent the cumulative excess risk at each unit of time and are useful to assess changes over time graphically(41). Confidence intervals above zero for a concrete age indicate a significant risk, below zero indicate a protective effect, and confidence intervals including zero show a non-significant risk(42). Models were adjusted for sex, age, marital status, level of education, household wealth, region, vigorous physical activity, tobacco consumption, HAT, depression, and presence of NCDs, all as time-varying covariates. The interaction between COPD and HAT was also assessed. Age was used as the time measure. Participants who were alive at the end of the study period or in their final assessment were censored. In the modeling process, data were left-truncated because we considered the first interview as the time of diagnosis in the participants whom and NCD had been diagnosed before baseline. All analyses were performed using R Version 4.0.3.(43). Statistical significance was set at $p<0.05$.

Results

We identified three HAT in each of the three birth cohorts according to lower BIC and the sample sizes not lower than 5% (Supplementary Table 2 and Supplementary Figure 1). Although four trajectories met the selection criteria in the oldest birth cohort, we decided to select a three-trajectory model to facilitate comparison between the three cohorts. In all birth cohorts, the trajectories were parallel. The first trajectory group included individuals with the highest scores on the healthy ageing scale and the third with the worse scores. We named each trajectory group as “high”, “medium”, and “low”, respectively.

Table 1 shows the characteristics of participants. Those participants of the oldest group (born ≤1935) showed a higher prevalence of COPD (12.50%), followed by those born between 1935 and 1945 (9.57%), ($p<0.001$). The oldest group presented lower proportions of the “high” HAT (31.60%) compared with the other two birth cohorts ($p<0.001$). Finally, the proportion of

For peer review only - http://bmjopen.bmj.com/site/about/guidelines.xhtml

For peer review only

deaths increased with age, being lower in the >1945 (2.07%) and higher in the ≤1935 sub-sample (16.90%) (p<0.001).

Table 1. Main characteristics of the sample broken down by year of birth

Characteristics	Years of birth cohort			*p* value[1]
	≤1935 (N=9738)	1936-1945 (N=9254)	>1945 (N=9865)	
**Female, n (%)**	5407 (55.50)	4879 (52.70)	5382 (54.60)	<0.001
**Age, mean (SD)**	74.40 (5.94)	61.30 (2.95)	52.10 (2.58)	<0.001
**Marital status, n (%)**				<0.001
Single	452 (4.64)	468 (5.06)	638 (6.47)	
Married	5832 (59.90)	7343 (79.30)	8051 (81.60)	
Divorced	338 (3.47)	603 (6.52)	950 (9.63)	
Widowed	3646 (37.40)	1203 (13.00)	531 (5.38)	
**Education level, n (%)**				<0.001
Less than primary	994 (10.20)	417 (4.51)	246 (2.49)	
Primary	3895 (40.00)	2513 (27.20)	1604 (16.30)	
Secondary	3734 (38.30)	4570 (49.40)	5520 (56.00)	
Tertiary	1115 (11.40)	1754 (19.00)	2495 (25.30)	
**Wealth quintiles, n (%)**				<0.001
1st (worst)	1798 (18.50)	801 (8.66)	687 (6.96)	
2nd	2479 (25.50)	1380 (14.90)	960 (9.73)	
3rd	2185 (22.40)	1895 (20.50)	1481 (15.00)	
4th	1721 (17.70)	2247 (24.30)	2494 (25.30)	
5th (best)	1555 (16.00)	2931 (31.70)	4243 (43.00)	
**Region, n (%)**				0.049
Northern Europe	1557 (16.00)	1483 (16.00)	1473 (14.90)	
Western Europe	4842 (49.70)	4676 (50.50)	5090 (51.60)	
Southern Europe	3339 (34.30)	3095 (33.40)	3302 (33.50)	
**Healthy ageing trajectories, n (%)**				<0.001
High	3073 (31.60)	4620 (49.90)	5962 (60.40)	
Medium	4713 (48.40)	3491 (37.70)	3050 (30.90)	
Low	1952 (20.00)	1143 (12.40)	853 (8.65)	
**Physical activity, n (%)**	4934 (50.70)	7107 (76.80)	8283 (84.00)	<0.001
**Ever smoked, n (%)**	3910 (40.20)	4572 (49.40)	5543 (56.20)	<0.001
**Diseases, n (%)**				
Diabetes	1821 (18.70)	1628 (17.60)	1132 (11.50)	<0.001
Hypertension	5334 (54.80)	4647 (50.20)	3500 (35.50)	<0.001
Joint disorders	3914 (40.20)	3018 (32.60)	2407 (24.40)	<0.001
Asthma	703 (7.22)	550 (5.94)	497 (5.04)	<0.001
COPD	1214 (12.50)	886 (9.57)	622 (6.31)	<0.001
Myocardial infarction	3022 (31.00)	1751 (18.90)	957 (9.70)	<0.001

For peer review only

Stroke	1119 (11.50)	565 (6.11)	332 (3.37)	<0.001
Depression	4340 (44.60)	3387 (36.60)	3444 (34.90)	<0.001
**Death, n (%)**	1642 (16.90)	451 (4.87)	204 (2.07)	<0.001

Note. Household income was divided into 5 quintiles (the first indicating the lowest income). Marital status "married" category included "currently married or cohabiting", and "divorced" included "divorced or separated". Abbreviations: COPD, chronic obstructive pulmonary disease. [1]Based on T-tests for numerical variables and Chi-square tests for categorical variables.

Three Aalen regression models were conducted: one with only the variable COPD, the second with only the HAT variable, and the third with COPD and the HAT adjusted for covariates. The estimated cumulative coefficients of the first and second model are presented in Supplementary Figure 2, and those from the third model are presented in Figure 1, according to the >1945, 1936-1945, and ≤1935 sub-samples, respectively. In the first model, COPD diagnosis had a significant risk on mortality in the three birth cohort groups: from 74 years old onwards in the ≤1935 sub-sample, from 65 years old onwards in the other two sub-samples (see Supplementary Figure 2). In the second model, regarding the HAT, those individuals classified in "low" trajectories had a significant risk of mortality: in the ≤1935 sub-sample, there was a significant risk of death from 76 to 94 years, and from 97 to 98; in the 1936-1945 sub-sample from 63 onwards; and in the >1945 sub-sample from 60 onwards. Those following a "medium" HAT had a significant risk of death in the ≤1935 sub-sample intermittently from 76 to 86 years and at 98 years; in the 1936-1945 sub-sample from 65 onwards; and in the >1945 sub-sample from 62 onwards (see Supplementary Figure 2).

Figure 1 shows the estimated cumulative coefficients calculated from the third model (including all variables) for each birth cohort. In this model, although the risk effect of COPD increases across age, it was rather non-significant (only a small effect in the ≤1935 sub-sample around 76 and 77 years old). In the case of the HAT, "low" trajectories were associated with a higher risk of mortality in the case of the ≤1935 sub-sample (from 88 to 90). There was a significant mortality risk in the 1936-1945 and the >1945 birth cohorts (from 71 onwards and 60 onwards, respectively). "Medium" HAT had only a significant effect in the 1936-1945 sub-

For peer review only - http://bmjopen.bmj.com/site/about/guidelines.xhtml

For peer review only

sample, from 74 onwards. The interaction between COPD and HAT was assessed in the third model. A significant effect was only found in the model with the ≤1935 sub-sample. The interaction showed a significant effect (higher risk of death) for participants with COPD and a “low” HAT, with the highest risk of death at the age of 75 and from 81 to 87.

Completed and detailed results of the fitting of Aalen’s additive regression models are presented in Supplementary Figure 3.

Discussion

We analysed the association of COPD with the risk of mortality and the moderating role of HAT in the SHARE study, a population-based cohort of middle-aged and older adults from 12 European countries who were followed up for nine years. To account for potential cohort effects, we analysed the results separately in three groups: those born after 1945 (aged 50+), born between 1936 and 1945 (ages from 58 to 70 years old), and born in 1935 or earlier (ages from 69 to 104 years old).

Our findings show that COPD increased the risk of mortality in the three birth cohorts. However, this association was no longer significant after adjusting for demographic and economic variables, presence of other NCDs and depression, and HAT. In line with previous research, the study of mortality in COPD patients is quite cumbersome, and multiple variables may play a role in this association. For example, lung cancer and COPD mortality were assessed, including several variables (residential characteristics, marital status, education, health insurance, and family income) in a research study based on The National Longitudinal Mortality Study in the United States(44). They found that COPD mortality rates were highest among 65 to 74 years old, in males and non-Hispanic whites(44). The results concerning the periods are consistent with those we found before adjustment, suggesting the existence of a period of increased risk of mortality in COPD patients. In another study based on The National

For peer review only - http://bmjopen.bmj.com/site/about/guidelines.xhtml

For peer review only

Mortality Database of Statistics Canada, the mortality related to COPD varied by age, sex, birth cohort, and the province(45). In that study, the mortality risk attributed to COPD decreased in male and female cohorts born after 1920 to 1924, whereas between 1971 and 1983 the mortality ratios were stable(45). Thus, performing the analyses considering different birth cohorts seems appropriate since exposure to risk factors for COPD such as tobacco consumption or occupational pollution might greatly vary across birth cohorts. Moreover, previous studies on the risk of COPD mortality have reported differences in age, sex, birth cohort, location, household income, education, and marital status(44–47). Thus, the study of mortality associated with COPD needs to account for the potential confounding effects of these risk factors.

One potential confounder is the region of residence, as indicated in previous studies(48). Despite not being the focus of our study, we identify that living in Western or Southern Europe had a protective effect on the risk of all-cause mortality, compared to Northern Europe (Denmark and Sweden). Similarly, Blanco et al. (2017) found a lower mean COPD prevalence in Southern Europe (10.8%) compared to Northern Europe (11.5%). However, variations in COPD prevalence were also found among countries of the same European region(12). In Northern Europe, it was higher in Denmark (ranging from 12% to 25%) than in Sweden (ranging from 2% to 20%); whereas in Southern Europe, Italy showed higher prevalence (ranging from 12% to 23%), than in Spain (from 7 to 10%)(12). The greater COPD prevalence and its associated mortality risk in Denmark could be a consequence of a very high smoking prevalence in the past five decades, resulting in the highest COPD prevalence in the western world(49). This heterogeneity among countries and regions might suggest the need for a better understanding of the underlying mechanisms.

Regarding the HAT, our results seem to confirm that participants (from different birth cohorts) with "low" and "medium" HAT (i.e., worse health status) have a higher risk of mortality

For peer review only

compared to those classified into “high” HAT. This effect remains after adjusting for covariates, although in the case of the “medium” trajectories, only a significant effect was found in the 1936-1945 birth cohort (constituted by people aged 58 to 70 years old). According to our results, “low” trajectories seem to discriminate a poorer health status in a better way and to predict mortality, even after adjusting for confounders. Previous studies examined the connection between healthy ageing and mortality, albeit using different indicators(50,51). In a South Brazilian population-based cohort, researchers differentiated between normal ageing and successful ageing (defined as a good state of health, a complete absence of functional disability and mood changes, and no cognitive impairment)(50). They detected that successful agers had lower mortality rates, and the normal agers had a higher risk for mortality(50). These results may be extrapolated to our “low” and “high” HAT, being the last the equivalent to “successful ageing”. Another study used The Healthy Ageing Index (HAI) as a summary measure of physiologic ageing(51), composed of cardiovascular, lung, cognitive, metabolic, and kidney function markers. In that study, HAI scores tended to increase with age (meaning worse healthy ageing) and predicted mortality from a given time-point(51). Hence, composite measures of ageing seem to be powerful tools to predict mortality and identify individuals at a higher risk.

One of the main results from our study is that the association between COPD and risk of mortality depended upon the HAT of the oldest participants (i.e., born ≤1935). Individuals with COPD and a “low” trajectory of healthy ageing were more likely to die at the age of 75 years old and from 81 to 87, compared with people with COPD and a “medium” or “high” HAT. The healthy ageing scale covers several domains (vitality, sensory skills, mobility, cognition, and ADL/IADL) and could negatively affect those patients with worse COPD symptoms(7–10). The fact that these results were found only in the oldest sub-sample may be related to the course of the disease since COPD is a progressive disease, and exacerbations and hospitalisations are particularly common among older individuals(52). Our results point out temporary spaces

For peer review only - http://bmjopen.bmj.com/site/about/guidelines.xhtml

where older COPD patients with a "low" HAT are at higher risk of mortality. Thus, future efforts should be concentrated on those aged 75 years old and from 81 to 87.

Few studies have analysed the relationship between health status in COPD patients to the best of our knowledge. These studies were based on self-reported perceived health status assessed through the SF-12 questionnaire, a generic instrument to evaluate physical and mental health(53,54). The main finding in one of these studies that used data from the BOLD project was that COPD severity was an important determinant of health status (more severity linked to poorer health status)(53). Although these studies considered the health status of people with COPD, we have not found any study that used a composite measure of healthy ageing as we have done. An integrated measure assessing intrinsic capacity and functional ability could be a useful tool in daily clinical practice for patient prognosis, as well as a mortality predictor, and for the creation of future public health strategies addressing COPD patients' needs(22). While it is true that other composite tools to predict COPD mortality are available (such as St George's Respiratory Questionnaire(55), or the BODE index(56)), the healthy ageing scale is a comprehensive tool that could be applied not only to COPD patients but also to patients with multimorbidity.

Strengths and limitations

These findings should be interpreted in light of the following limitations. Firstly, the presence or absence of COPD and NCDs was based on self-reported diagnostics. Thus they might be affected by measurement errors. Nevertheless, some authors sustain self-reported diagnostics as a well-established method for measuring NCDs in population-based studies(57). Secondly, we made some assumptions in terms of age of diagnosis. Due to the high percentage of missingness (48%) in the age of the first NCD diagnosis, we selected the age of the earliest diagnosis of each NCD within the five waves. That is, we coded the age of the participant in the wave he/she reported the first time having some of the included diseases. Despite being an

For peer review only - http://bmjopen.bmj.com/site/about/guidelines.xhtml

assumption, there are only two years between each wave in the SHARE study. Thus, we believe that there is not a significant impact on our conclusions. Thirdly, we split the sample into three birth cohorts when performing the analyses, and we reported the mortality risk in each group. By doing so, we captured potential cohort effects which people from different birth cohorts can be influenced by different exposure to COPD-related risk factors that contribute differently to mortality, as the different trends in smoking prevalence. For each birth cohort, the survival analysis can be focused according to the years of the interview or according to the age of the participants. We finally decided to do it according to the age of the participants because working with time-varying variables and without the assumption of proportional risks, the fluctuations in mortality risk according to age could be better interpreted. However, this introduces a problem of left truncation since the age range observed for each participant is different, although we took this into account in the additive regression model. Fourthly, another issue is that the age range observed for each birth cohort is also different so that the excess cumulative risk curve starts at the first observed age. Therefore, the bias of the healthy participant in the first wave of the study means that there is no significant excess risk in the first ages of observation. Fifthly, we considered several variables that could affect mortality in COPD patients, such as the presence of other NCDs, ever smoked, the practice of vigorous physical activity and the role of healthy ageing trajectories on mortality risk. However, other known factors with cumulative effects on COPD, such as long-term smoking and physical activity or lung function data (52), were not available in the study. Thus we could not control for their potential confounding effect. Alongside these limitations, this study had some strengths. Firstly, the analyses were performed in different birth cohorts (>1945, 1936-1945, and ≤1935) to assess differences in mortality risks related to societal changes, such as lifestyle behaviours and occupation trends. Secondly, we used a novel measurement scale of healthy ageing, including intrinsic capacity and functional ability variables. Compared with the use of different health indicators separately, we believe that using an integrated and reliable

For peer review only - http://bmjopen.bmj.com/site/about/guidelines.xhtml

For peer review only

measure of health status is a powerful tool to predict the mortality risk of the participants. Thirdly, the calculation of Aalen additive hazards models rather than Cox models allowed the inclusion of time-variant variables in the analyses.

Conclusion

COPD is a costly and preventable disease that has large-scale implications for patients' quality of life and society in general(58,59). Our findings suggest that the association between COPD and the risk of mortality in the general population of middle-aged and older adults might be explained by the presence of other risk factors. However, for older people with COPD (i.e., aged 69 or older), having a poor trajectory of healthy ageing might compromise their survival. Especial attention should be paid to these patients, with the healthy ageing scale as a suitable tool identifying older patients with COPD at high risk of mortality(21).

Competing interests

The authors declare no conflict of interest.

Ethics statements

Patient consent for publication

Not required.

Ethics approval

Ethical approvals for waves from 1 to 3 were granted by the Ethics Committee of the University of Mannheim. For waves 4 and 5, the SHARE projects were reviewed and approved by the Ethics Council of the Max-Planck Society. All data were anonymised and EHR confidentially was respected in accordance with national and international law.

Data sharing statement: The original data of the Survey of Health, Ageing and Retirement in Europe – SHARE is available on the official website (http://www.share-project.org/home0.html). R codes for harmonising the healthy ageing scale is available on https://athlos.pssjd.org/study/share-hs.

For peer review only - http://bmjopen.bmj.com/site/about/guidelines.xhtml

Funding

This work was supported by the five-year Ageing Trajectories of Health: Longitudinal Opportunities and Synergies (ATHLOS) project. The ATHLOS project has received funding from the European Union's Horizon 2020 research and innovation program under grant agreement No 635316. BO's work is supported by the Miguel Servet Program (CP20/00040), funded by Instituto de Salud Carlos III and co-funded by European Union (ERDF/ESF, "Investing in your future"). DF's work has been supported by Marsden grant E2987-3648 administrated by the Royal Society of New Zealand) and by grant 2017 SGR 622 (GRBIO) administrated by the Departament d'Economia i Coneixement de la Generalitat de Catalunya (Spain).

Acknowledgements

The SHARE study is funded by the European Commission through FP5 (QLK6-CT-2001-00360), FP6 (SHARE-I3: RII-CT-2006-062193, COMPARE: CIT5-CT-2005-028857, SHARELIFE: CIT4-CT-2006-028812) and FP7 (SHARE-PREP: N°211909, SHARE-LEAP: N°227822, SHARE M4: N°261982). Additional funding from the German Ministry of Education and Research, the Max Planck Society for the Advancement of Science, the U.S. National Institute on Aging (U01_AG09740-13S2, P01_AG005842, P01_AG08291, P30_AG12815, R21_AG025169, Y1-AG-4553-01, IAG_BSR06-11, OGHA_04-064, HHSN271201300071C) and from various national funding sources is gratefully acknowledged (see www.share-project.org).

Author contributions

IB: Participated in database management, drafted the paper, carried out the statistical analyses and worked on the interpretation of data. She also gave final approval of the version to be published and agreed to be accountable for all aspects of the work in ensuring that questions related to the accuracy or integrity of any part of the work are appropriately investigated and resolved; **AS:** Participated in the study design, database management, carried out the statistical analyses, gave statistical support and critical revision of the paper. He also gave final approval of the version to be published and agreed to be accountable for all aspects

For peer review only

For peer review only - http://bmjopen.bmj.com/site/about/guidelines.xhtml

of the work in ensuring that questions related to the accuracy or integrity of any part of the work are appropriately investigated and resolved; **DF:** Participated in the statistical support and critical revision of the paper. He also gave final approval of the version to be published and agreed to be accountable for all aspects of the work in ensuring that questions related to the accuracy or integrity of any part of the work are appropriately investigated and resolved; **JMH:** Participated in the acquisition of data, and critical revision of the paper. He also gave final approval of the version to be published and agreed to be accountable for all aspects of the work in ensuring that questions related to the accuracy or integrity of any part of the work are appropriately investigated and resolved; **BO:** Participated in the critical revision of the paper. She also gave final approval of the version to be published and agreed to be accountable for all aspects of the work in ensuring that questions related to the accuracy or integrity of any part of the work are appropriately investigated and resolved.

References

1. Miniati M, Monti S, Pavlickova I, Bottai M. Survival in COPD: impact of lung dysfunction and comorbidities. Med. 2014;93(12):e76.

2. Okely JA, Shaheen SO, Weiss A, Gale CR. Wellbeing and chronic lung disease incidence: the Survey of Health, Ageing and Retirement in Europe. Leroyer C, editor. PLoS One [Internet]. 2017 Jul 20 [cited 2020 May 19];12(7):e0181320. Available from: https://dx.plos.org/10.1371/journal.pone.0181320

3. World Health Organization. World Health Statistics 2008 [Internet]. 2008 [cited 2020 May 20]. Available from: https://www.who.int/whosis/whostat/EN_WHS08_Full.pdf?ua=1

4. Buist AS, McBurnie MA, Vollmer WM, Gillespie S, Burney P, Mannino DM, et al. International variation in the prevalence of COPD (The BOLD Study): a population-based prevalence study. Lancet. 2007 Sep 1;370(9589):741–50.

5. Duan R-R, Hao K, Yang T. Air pollution and chronic obstructive pulmonary disease. Chronic Dis Transl Med. 2020 Dec 1;6(4):260–9.

6. Rosenberg SR, Kalhan R, Mannino DM. Epidemiology of chronic obstructive pulmonary disease: prevalence, morbidity, mortality, and risk factors. Semin Respir Crit Care Med. 2015 Aug 5;36(4):457–69.

7. Bourbeau J. Activities of Life: the COPD patient. COPD. 2009;6(3):192–200.

8. Barusso MS, Gianjoppe-Santos J, Basso-Vanelli RP, Regueiro EM, Panin JC, Lorenzo VAP Di. Limitation of activities of daily living and quality of life based on COPD combined classification. Respir Care. 2015 Mar 1;60(3):388–98.

For peer review only - http://bmjopen.bmj.com/site/about/guidelines.xhtml

9. Medina-Mirapeix F, Bernabeu-Mora R, Piedad Sánchez-Martínez M, Montilla-Herrador J, Bernabeu-Mora M, Escolar-Reina P. Mobility limitations related to reduced pulmonary function among aging people with chronic obstructive pulmonary disease. PLoS One. 2018 May 1;13(5):e0196152.

10. Dodd JW. Lung disease as a determinant of cognitive decline and dementia. Alzheimer's Res Ther. 2015;7(1):1–8.

11. Eroglu SA, Gunen H, Yakar HI, Yildiz E, Kavas M, Duman D. Influence of comorbidities in long-term survival of chronic obstructive pulmonary disease patients. J Thorac Dis. 2019 Apr 1;11(4):1379–86.

12. Blanco I, Diego I, Bueno P, Fernández E, Casas-Maldonado F, Esquinas C, et al. Geographical distribution of COPD prevalence in Europe, estimated by an inverse distance weighting interpolation technique. Int J Chron Obs Pulmon Dis [Internet]. 2017 [cited 2020 Dec 9];13:57–67. Available from: /pmc/articles/PMC5743112/?report=abstract

13. Menezes AMB, Perez-Padilla R, Jardim JRB, Muiño A, Lopez MV, Valdivia G, et al. Chronic obstructive pulmonary disease in five Latin American cities (the PLATINO study): A prevalence study. Lancet. 2005 Nov 26;366(9500):1875–81.

14. Blanco I, Diego I, Bueno P, Fernández E, Casas-Maldonado F, Esquinas C, et al. Geographical distribution of COPD prevalence in Europe, estimated by an inverse distance weighting interpolation technique. Int J Chron Obs Pulmon Dis. 2017;13:57–67.

15. Nowak D, Berger K, Lippert B, Kilgert K, Caeser M, Sandtmann R. Epidemiology and health economics of COPD across Europe: A critical analysis. Vol. 4, Treatments in Respiratory Medicine. Treat Respir Med; 2005. p. 381–95.

16. Lortet-Tieulent J, Soerjomataram I, López-Campos JL, Coebergh JW, Ancochea J, Soriano JB. International trends in COPD mortality, 1995-2017. Eur Respir J. 2019 Dec 1;54(6):1901791.

17. Davies Adeloye, Stephen Chua, Chinwei Lee, Catriona Basquill, Angeliki Papana, Evropi Theodoratou, Harish Nair, Danijela Gasevic, Devi Sridhar, Harry Campbell, Kit Yee Chan, Aziz Sheikh, Igor Rudan and GHERG (GHERG). Global and regional estimates of COPD prevalence: Systematic review and meta–analysis. J Glob Health. 2015;5(2):020415.

18. Pinto-Plata VM, Cote C, Cabral H, Taylor J, Celli BR. The 6-min walk distance: Change over time and value as a predictor of survival in severe COPD. Eur Respir J [Internet]. 2004 Jan [cited 2021 Jan 8];23(1):28–33. Available from: https://pubmed.ncbi.nlm.nih.gov/14738227/

19. Spruit MA, Polkey MI, Celli B, Edwards LD, Watkins ML, Pinto-Plata V, et al. Predicting Outcomes from 6-Minute Walk Distance in Chronic Obstructive Pulmonary Disease. J Am Med Dir Assoc [Internet]. 2012 [cited 2021 Jan 8];13(3):291–7. Available from: https://pubmed.ncbi.nlm.nih.gov/21778120/

20. Puhan MA, Garcia-Aymerich J, Frey M, ter Riet G, Antó JM, Agustí AG, et al. Expansion of the prognostic assessment of patients with chronic obstructive pulmonary disease: the updated BODE index and the ADO index. Lancet [Internet]. 2009 Sep 4 [cited 2021 Jan 8];374(9691):704–11. Available from: https://pubmed.ncbi.nlm.nih.gov/19716962/

21. Puhan MA, Siebeling L, Zoller M, Muggensturm P, Riet G Ter. Simple functional

For peer review only

performance tests and mortality in COPD. Eur Respir J [Internet]. 2013 Oct 1 [cited 2021 Jan 8];42(4):956–63. Available from: http://ow.ly/mxrPx

22. Sanchez-Niubo A, Forero CG, Wu Y-T, Giné-Vázquez I, Prina M, De La Fuente J, et al. Development of a common scale for measuring healthy ageing across the world: results from the ATHLOS consortium. Int J Epidemiol [Internet]. 2020 Dec 4 [cited 2020 Dec 5];dyaa236:1–13. Available from: https://academic.oup.com/ije/advance-article/doi/10.1093/ije/dyaa236/6020095

23. Bui KL, Nyberg A, Maltais F, Saey D. Functional tests in chronic obstructive pulmonary disease, Part 1: Clinical relevance and links to the international classification of functioning, disability, and health. In: Annals of the American Thoracic Society [Internet]. American Thoracic Society; 2017 [cited 2021 Jan 8]. p. 778–84. Available from: http://www.atsjournals.org/doi/10.1513/AnnalsATS.201609-733AS

24. Sanchez-Niubo A, Egea-Cortés L, Olaya B, Caballero FF, Ayuso-Mateos JL, Prina M, et al. Cohort profile: the ageing trajectories of health - longitudinal ppportunities and synergies (ATHLOS) project. Int J Epidemiol. 2019;48(4):1052-1053i.

25. Börsch-Supan A, Brandt M, Hunkler C, Kneip T, Korbmacher J, Malter F, et al. Data resource profile: The survey of health, ageing and retirement in europe (SHARE). Int J Epidemiol. 2013;42(4):992–1001.

26. Bergmann, Michael; Kneip, Thorsten; De Luca, Giuseppe; Scherpenzeel A. Survey Participation in the Survey of Health, Ageing and Retirement in Europe (SHARE), Wave 1-6. Based on Release 6.0.0 (March 2017). Munich: Munich Center for the Economics of Aging (MEA); 2017.

27. Wolfrum R. Opinion of the ethics council of the Max Planck society on the “SHARE” project [Internet]. 2016. Available from: http://www.share-project.org/fileadmin/pdf_documentation/SHARE_ethics_approvals.pdf

28. Bergmann, M. Kneip, T., De Luca, G., & Scherpenzeel A. Survey participation in the Survey of Health, Ageing and Retirement in Europe (SHARE), Wave 1-7. Based on Release 7.0.0. Munich: SHARE-ERIC; 2019.

29. Sanchez-Niubo A, Egea-Cortés L, Olaya B, Caballero FF, Ayuso-Mateos JL, Prina M, et al. Cohort Profile: The Ageing Trajectories of Health - Longitudinal Opportunities and Synergies (ATHLOS) project. Int J Epidemiol. 2019 Aug 1;48(4):1052-1053I.

30. World Health Organization. World report on ageing and health 2015 [Internet]. 2015 [cited 2020 Jul 27]. Available from: https://www.who.int/ageing/events/world-report-2015-launch/en/

31. Berlin KS, Parra GR, Williams NA. An Introduction to Latent Variable Mixture Modeling (Part 2): Longitudinal Latent Class Growth Analysis and Growth Mixture Models. J Pediatr Psychol. 2014 Mar 1;39(2):188–203.

32. Reischies F, Lobo A, Turrina C, Jonker C, Fuhrer R, Fichter M, et al. Development of the EURO–D scale – a European Union initiative to compare symptoms of depression in 14 European centres. Br J Psychiatry. 2008;174(04):330–8.

33. Copeland JRM, Beekman ATF, Dewey ME, Hooijer C, Jordan A, Lawlor BA, et al. Depression in Europe. Geographical distribution among older people. Br J Psychiatry [Internet]. 1999 [cited 2020 Dec 2];174(APR.):312–21. Available from: https://pubmed.ncbi.nlm.nih.gov/10533550/

For peer review only - http://bmjopen.bmj.com/site/about/guidelines.xhtml

34. WHO. Country groupings [Internet]. Available from: https://www.who.int/quantifying_ehimpacts/global/ebdcountgroup/en/ (7 February 2019, date last accessed)

35. United Nations Statistical Division (UNSD). Countries or areas/geographical regions [Internet]. Available from: https://unstats.un.org/unsd/methodology/m49/ (7 February 2019, date last accessed)

36. Tein JY, Coxe S, Cham H. Statistical power to detect the correct number of classes in latent profile analysis. Struct Equ Model. 2013;20(4):640–57.

37. Nylund-Gibson K, Choi AY. Ten frequently asked questions about latent class analysis. Transl Issues Psychol Sci. 2018;4(4):440–61.

38. Aalen O. A Model for Nonparametric Regression Analysis of Counting Processes. In Springer, New York, NY; 1980 [cited 2020 Dec 5]. p. 1–25. Available from: https://link.springer.com/chapter/10.1007/978-1-4615-7397-5_1

39. Aalen OO. A linear regression model for the analysis of life times. Stat Med [Internet]. 1989 [cited 2020 Dec 5];8(8):907–25. Available from: https://pubmed.ncbi.nlm.nih.gov/2678347/

40. O. O. Aalen; O. Borgan; H. K. Gjessing. Survival and event history analysis: a process point of view. Springer. New York; 2008.

41. Xie X, Strickler HD, Xue X. Additive hazard regression models: An application to the natural history of human papillomavirus. Vol. 2013, Computational and Mathematical Methods in Medicine. 2013.

42. Aalen OO, Scheike TH. Aalen's Additive Regression Model. In: Encyclopedia of Biostatistics [Internet]. Chichester, UK: John Wiley & Sons, Ltd; 2005 [cited 2021 Feb 2]. Available from: http://doi.wiley.com/10.1002/0470011815.b2a11002

43. Team RC. R: A language and environment for statistical computing [Internet]. Vienna, Austria: R Foundation for Statistical Computing; 2020. Available from: https://www.r-project.org/

44. Lewis DR, Clegg LX, Johnson NJ. Lung disease mortality in the United States: the National Longitudinal Mortality Study. Int J Tuberc Lung Dis [Internet]. 2009 Aug [cited 2021 Feb 4];13(8):1008–14. Available from: /pmc/articles/PMC2765862/?report=abstract

45. Manfreda J, Mao Y, Litven W. Morbidity and mortality from chronic obstructive pulmonary disease. Am Rev Respir Dis [Internet]. 1989 [cited 2021 Jan 20];140(3):16–26. Available from: https://pubmed.ncbi.nlm.nih.gov/2782756/

46. Ntritsos G, Franek J, Belbasis L, Christou MA, Markozannes G, Altman P, et al. Gender-specific estimates of COPD prevalence: a systematic review and meta-analysis. Int J Chron Obstruct Pulmon Dis [Internet]. 2018 May 10 [cited 2020 Dec 9];13:1507–14. Available from: /pmc/articles/PMC5953270/?report=abstract

47. Hummer RA, Hernandez EM. The effect of educational attainment on adult mortality in the United States. Popul Bull [Internet]. 2013 Jun [cited 2020 Dec 9];68(1):1–16. Available from: http://www.ncbi.nlm.nih.gov/pubmed/25995521

48. OECD/European Union. Mortality from respiratory diseases. In: OECD Publishing, editor. Health at a Glance: Europe 2018: State of Health in the EU Cycle. Paris/European Union,

For peer review only - http://bmjopen.bmj.com/site/about/guidelines.xhtml

Brussels; 2018.

49. Lange P, Tøttenborg SS, Sorknæs AD, Andersen JS, Søgaard M, Nielsen H, et al. Danish register of chronic obstructive pulmonary disease. Clin Epidemiol [Internet]. 2016 Oct 25 [cited 2020 Dec 9];8:673–8. Available from: /pmc/articles/PMC5094652/?report=abstract

50. Camozzato AL, Godinho C, Chaves MLF. Effect of successful aging on mortality in older individuals: the PALA study. Dement e Neuropsychol [Internet]. 2014 [cited 2021 Feb 4];8(2):182–6. Available from: /pmc/articles/PMC5619127/?report=abstract

51. O'Connell MDL, Marron MM, Boudreau RM, Canney M, Sanders JL, Kenny RA, et al. Mortality in relation to changes in a healthy aging index: the Health, Aging, and Body Composition Study. Journals Gerontol - Ser A Biol Sci Med Sci [Internet]. 2019 May 1 [cited 2021 Feb 4];74(5):726–32. Available from: https://pubmed.ncbi.nlm.nih.gov/29733331/

52. Mannino DM. COPD: epidemiology, prevalence, morbidity and mortality, and disease heterogeneity. Chest [Internet]. 2002 [cited 2021 Feb 4];121(5):121–6. Available from: https://pubmed.ncbi.nlm.nih.gov/12010839/

53. Janson C, Marks G, Buist S, Gnatiuc L, Gislason T, McBurnie MA, et al. The impact of COPD on health status: Findings from the BOLD study. Eur Respir J [Internet]. 2013 Dec 1 [cited 2021 Feb 4];42(6):1472–83. Available from: http://ow.ly/p1clxwww.erj.ersjournals.com

54. López Varela MV, Montes de Oca M, Halbert R, Muiño A, Tálamo C, Pérez-Padilla R, et al. Comorbidities and Health Status in Individuals With and Without COPD in Five Latin American Cities: The PLATINO Study. Arch Bronconeumol [Internet]. 2013 Nov 1 [cited 2021 Feb 4];49(11):468–74. Available from: https://www.archbronconeumol.org/en-comorbidities-health-status-in-individuals-articulo-S1579212913001729

55. Jones PW, Quirk FH, Baveystock CM. The St George's Respiratory Questionnaire. Respir Med [Internet]. 1991 [cited 2021 Jan 20];85:25–31. Available from: https://pubmed.ncbi.nlm.nih.gov/1759018/

56. Celli BR, Cote CG, Marin JM, Casanova C, Montes de Oca M, Mendez RA, et al. The Body-Mass Index, Airflow Obstruction, Dyspnea, and Exercise Capacity Index in Chronic Obstructive Pulmonary Disease. N Engl J Med [Internet]. 2004 Mar 4 [cited 2021 Jan 20];350(10):1005–12. Available from: https://pubmed.ncbi.nlm.nih.gov/14999112/

57. Huntley AL, Johnson R, Purdy S, Valderas JM, Salisbury C. Measures of multimorbidity and morbidity burden for use in primary care and comminuty settings: a systematic review and guide. Ann Fam Med [Internet]. 2012;10(2):134–41. Available from: https://www.samsungrecycle.co.uk/

58. Chapmann KR, Mannino DM, Soriano JB, Vermeire PA, Buist AS, Thun MJ, et al. Epidemiology and costs of chronic obstructive pulmonary disease [Internet]. Vol. 27, European Respiratory Journal. European Respiratory Society; 2006 [cited 2020 Dec 9]. p. 188–207. Available from: https://erj.ersjournals.com/content/27/1/188

59. Quaderi SA, Hurst JR. The unmet global burden of COPD [Internet]. Vol. 3, Global Health, Epidemiology and Genomics. Cambridge University Press; 2018 [cited 2020 Dec 9]. p. 1–3. Available from: https://www.ncbi.nlm.nih.gov/pmc/articles/PMC5921960/

For peer review only - http://bmjopen.bmj.com/site/about/guidelines.xhtml

For peer review only

Figure 1. Cumulative excess risk of mortality associated with COPD, HAT by birth cohort and their interaction in the oldest birth cohort.

Note: All models were adjusted by sex, education, wealth, marital status, region, depression, diabetes, hypertension, joint disorders, asthma, myocardial infarction, and stroke.

For peer review only

For peer review only - http://bmjopen.bmj.com/site/about/guidelines.xhtml

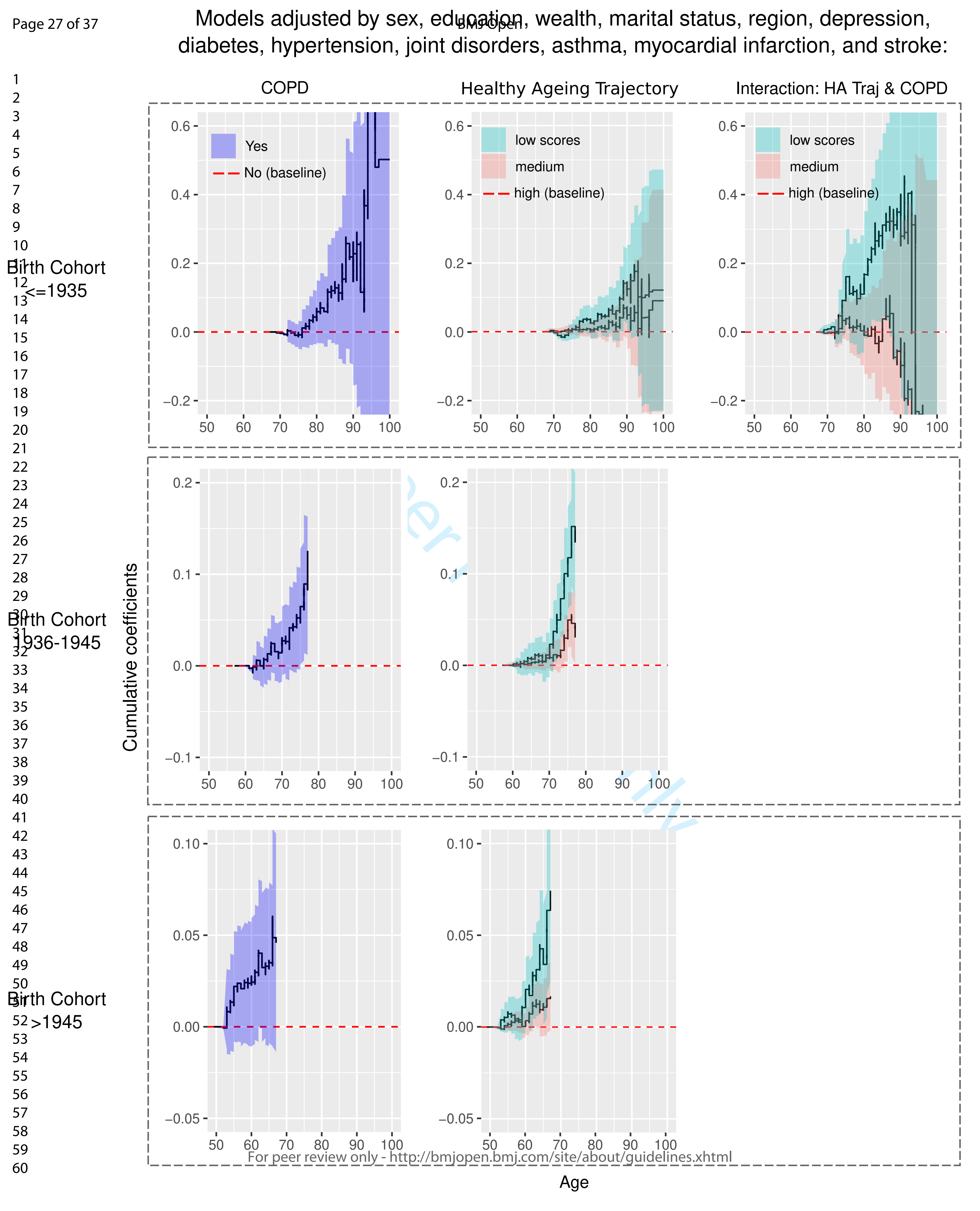
Models adjusted by sex, education, wealth, marital status, region, depression, diabetes, hypertension, joint disorders, asthma, myocardial infarction, and stroke:
COPD
Healthy Ageing Trajectory
Interaction: HA Traj & COPD
Yes
No (baseline)
low scores
medium
high (baseline)
Birth Cohort <=1935
Birth Cohort 1936-1945
Birth Cohort >1945
Cumulative coefficients
Age

Supplemental material

Supplementary Table 1. Harmonised items of the healthy ageing scale in the SHARE study

ATHLOS harmonized variables			SHARE
Memory	Self-reported ratings of memory at the time of the interview	Label	A4-B3 Memory self-rating
		Values	1 = None; 2 = Mild; 3 = Moderate; 4 = Severe; 5 = Extreme
		Harmonisation	1 = Absence; 2-5 = Presence
Immediate recall	Immediate recall of common nouns from a list	Label	ten words list learning first trial total
		Values	Continuous Variable 0-10
		Harmonisation	<=25% into Presence<br>>25% into Absence
Delayed recall	Test that assesses delayed recall using the common nouns from the list previously employed for measuring Immediate recall	Label	ten words list learning delayed recall total
		Values	Continuous Variable 0-10
		Harmonisation	<=25% into Presence<br>>25% into Absence
Verbal fluency†	Test that assesses verbal (semantic) fluency	Label	verbal fluency score
		Values	Continuous Variable 0-88
		Harmonisation	<=25% into Presence<br>>25% into Absence
Orientation in time	Difficulties for orientation in time, evaluated by a set of questions about the date and day of the week	Label	orientation to date, month, year and day of week
		Values	0-3 = bad; 4 = good
		Harmonisation	4 = Absence; 0-3 = Presence
Numeracy		Label	cf012_: Chance disease 10 perc. of 1000<br>cf013_: Half price [of a 300 Euro sofa]<br>cf014_: 6000 is two-thirds what is total price<br>cf015_: Amount in the savings account [on 2000 Euros after 2 years of 10% interest]
		Values	All: 1 = correct answer: 100; 2 = wrong answer: 10; 3 = wrong answer: 90; 4 = wrong answer: 900; 97 = wrong answer: Other answer keep 1 into 1; recode -1, 2, 3, 4, 5, 6 and 97 into 0
		Harmonisation	All 1 = Absence; Some 0 = Presence
Sleeping	Sleeping problems	Label	sleep (part of EURO-D)
		Values	0 = Not selected; 1 = Selected
		Harmonisation	0 = Absence; 1 = Presence
Pain	It measures if the participant experiences some degree of pain or if the participant does not present any pain at all	Label	bothered by: pain in back, knees, hips or other joint
		Values	0 = Not selected; 1 = Selected
		Harmonisation	0 = Absence; 1 = Presence
Energy	Self-reported high level of energy experienced at the time of the interview	Label	fatigue
		Values	1 = Yes; 5 = No
		Harmonisation	5 = Absence; 1 = Presence
		Label	bothered by: incontinence

For peer review only

Urine incontinence	It measures if the participant has experienced loss of urine (or has used any special device for urine leakage)	Values	0 = Not selected; 1 = Selected
		Harmonisation	0 = Absence; 1 = Presence
Near vision	Difficulties for near vision	Label	eyesight reading
		Values	1 = excellent; 2 = very good; 3 = good; 4 = fair; 5 = poor
		Harmonisation	1-3 = Absence; 4-5 = Presence
Far vision	Difficulties for far vision	Label	eyesight distance
		Values	1 = excellent; 2 = very good; 3 = good; 4 = fair; 5 = poor
		Harmonisation	1-3 = Absence; 4-5 = Presence
Eyesight	Difficulties in eye sight using glasses or corrective lens as usual	Label	Is your eyesight (using glasses or contact lens if you use them)
		Values	1 = excellent; 2 = very good; 3 = good; 4 = fair; 5 = poor; 6 = registered or legally blind
		Harmonisation	1-3 = Absence; 4-6 = Presence
Hearing in general	It measures if the participant experiences some difficulty for hearing (i.e., hearing someone talking on the other side of the room in a normal voice) or not, using a hearing aid as usual	Label	hearing
		Values	1 = excellent; 2 = very good; 3 = good; 4 = fair; 5 = poor
		Harmonisation	1-3 = Absence; 4-5 = Presence
Hearing in a conversation	It measures if the participant experiences some difficulty (including total disability) for following a conversation (i.e., if there is a background noise, or several people talking) or not, using a hearing aid as usual	Label	hearing with one person
		Values	1 = Yes; 5 = No
		Harmonisation	1 = Absence; 0= Presence
Stooping, kneeling or crouching	Difficulty for stooping, kneeling or crouching	Label	difficulties: stooping, kneeling, crouching
		Values	0 = Not selected; 1 = Selected
		Harmonisation	0 = Absence; 1 = Presence
Lifting or carrying weights	Difficulty for lifting or carrying weights	Label	difficulties: lifting or carrying weights over 5 kilos
		Values	0 = Not selected; 1 = Selected
		Harmonisation	0 = Absence; 1 = Presence
Climbing stairs	Difficulty for climbing stairs	Label	difficulties: climbing one flight of stairs
		Values	0 = Not selected; 1 = Selected
		Harmonisation	0 = Absence; 1 = Presence
Getting up	Difficulty for getting up from sitting down	Label	difficulties: getting up from chair
		Values	0 = Not selected; 1 = Selected
		Harmonisation	0 = Absence; 1 = Presence
Walking	Difficulty for walking by yourself and without using any special equipment	Label	difficulties: walking 100 metres
		Values	0 = Not selected; 1 = Selected
		Harmonisation	0 = Absence; 1 = Presence
Pulling or pushing	Difficulty for pulling or pushing large objects	Label	difficulties: pulling or pushing large objects
		Values	0 = Not selected; 1 = Selected
		Harmonisation	0 = Absence; 1 = Presence
Sitting	Difficulty for sitting for long periods	Label	difficulties: sitting two hours
		Values	0 = Not selected; 1 = Selected

For peer review only

		Harmonisation	0 = Absence; 1 = Presence
Reaching or extending arms	Difficulty for reaching / extending arms	Label	difficulties: reaching or extending arms above shoulder
		Values	0 = Not selected; 1 = Selected
		Harmonisation	0 = Absence; 1 = Presence
Walking speed	It is measured assessing the time that is taken to walk a distance	Label	walking speed
		Values	Continuous
		Harmonisation	<=25% into Presence<br>>25% into Absence
Dizziness	Dizziness problems when walking on a level surface	Label	bothered by: dizziness, faints or blackouts
		Values	0 = Not selected; 1 = Selected
		Harmonisation	0 = Absence; 1 = Presence
Picking up	Difficulty for picking up things with fingers, e.g. picking up a coin	Label	difficulties: picking up a small coin from a table
		Values	0 = Not selected; 1 = Selected
		Harmonisation	0 = Absence; 1 = Presence
Getting in or out of bed	Difficulty for getting in or out of bed	Label	difficulties: getting in or out of bed
		Values	0 = Not selected; 1 = Selected
		Harmonisation	0 = Absence; 1 = Presence
Bathing or showering	Difficulties for bathing or showering	Label	difficulties: bathing or showering
		Values	0 = Not selected; 1 = Selected
		Harmonisation	0 = Absence; 1 = Presence
Getting dressed	Difficulty for getting dressed	Label	difficulties: dressing, including shoes and socks
		Values	0 = Not selected; 1 = Selected
		Harmonisation	0 = Absence; 1 = Presence
Moving around the home	Difficulty for moving around the home	Label	difficulties: walking across a room
		Values	0 = Not selected; 1 = Selected
		Harmonisation	0 = Absence; 1 = Presence
Toilet	Difficulties for using the toilet	Label	difficulties: using the toilet, incl getting up or down
		Values	0 = Not selected; 1 = Selected
		Harmonisation	0 = Absence; 1 = Presence
Eating	Difficulties for eating	Label	difficulties: eating, cutting up food
		Values	0 = Not selected; 1 = Selected
		Harmonisation	0 = Absence; 1 = Presence
Housework	Difficulties for doing housework	Label	difficulties: doing work around the house or garden
		Values	0 = Not selected; 1 = Selected
		Harmonisation	0 = Absence; 1 = Presence
Shopping	Difficulties for shopping groceries	Label	difficulties: shopping for groceries
		Values	0 = Not selected; 1 = Selected
		Harmonisation	0 = Absence; 1 = Presence
Meals	Difficulties in preparing meals	Label	difficulties: preparing a hot meal
		Values	0 = Not selected; 1 = Selected
		Harmonisation	0 = Absence; 1 = Presence
Map	Difficulties for using a map	Label	difficulties: using a map in a strange place
		Values	0 = Not selected; 1 = Selected
		Harmonisation	0 = Absence; 1 = Presence
Money	Difficulties for managing money, bills, or expenses	Label	difficulties: managing money
		Values	0 = Not selected; 1 = Selected
		Harmonisation	0 = Absence; 1 = Presence
Medications	Difficulties for taking medications	Label	difficulties: taking medications

For peer review only

		Values	0 = Not selected; 1 = Selected
		Harmonisation	0 = Absence; 1 = Presence
Telephone	Difficulties for using telephone	Label	difficulties: telephone calls
		Values	0 = Not selected; 1 = Selected
		Harmonisation	0 = Absence; 1 = Presence

For peer review only

Supplementary Table 2. Results from the latent class growth analysis models for the three birth cohorts

≤1935								
**No. of classes**	**Loglik**	**npm**	**BIC**	**% class 1**	**% class 2**	**% class 3**	**% class 4**	**% class 5**
1	-83923.67	3	167875.0	100.00				
2	-80924.65	6	161904.6	51.39	48.60			
**3**	**-79920.76**	**9**	**159924.4**	**20.21**	**31.38**	**48.39**		
4	-79545.29	12	159201.1	7.37	32.59	23.22	36.80	
5	-79429.36	15	158996.8	31.64	23.08	1.31	7.19	36.76
**1936 – 1945**								
**No. of classes**	**Loglik**	**npm**	**BIC**	**% class 1**	**% class 2**	**% class 3**	**% class 4**	**% class 5**
1	-86218.80	3	172465.1	100.00				
2	-82811.31	6	165677.6	66.84	33.15			
**3**	**-81877.93**	**9**	**163838.3**	**49.72**	**12.44**	**37.83**		
4	-81611.24	12	163332.3	43.55	35.79	2.38	18.26	
5	-81540.03	15	163217.4	33.21	12.13	24.67	28.00	1.97
**>1945**								
**No. of classes**	**Loglik**	**npm**	**BIC**	**% class 1**	**% class 2**	**% class 3**	**% class 4**	**% class 5**
1	-89325.74	3	178679.1	100.00				
2	-85721.95	6	171499.2	23.37	76.62			
**3**	**-84856.16**	**9**	**169795.3**	**8.75**	**30.81**	**60.43**		
4	-84608.23	12	169327.1	30.97	53.19	13.34	2.47	
5	-84607.84	15	169354.0	2.39	13.17	32.36	52.02	0.02

Supplementary Figure 1. Trajectories of healthy ageing among birth cohorts

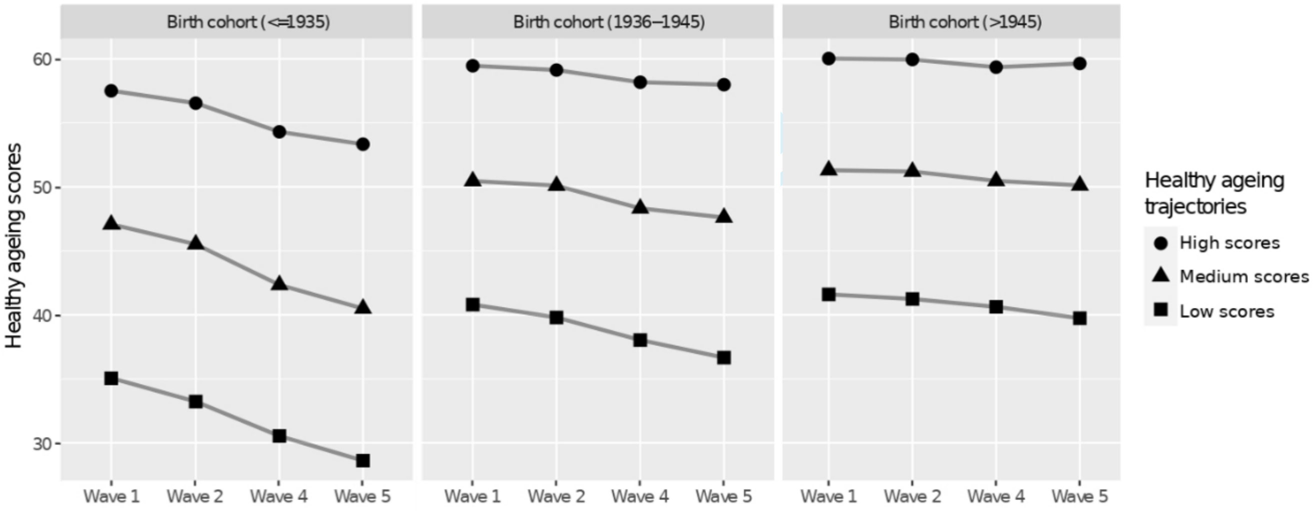

For peer review only - http://bmjopen.bmj.com/site/about/guidelines.xhtml

Supplementary Figure 2. Estimates of cumulative excess of risk of COPD and healthy ageing trajectories, separately by using the Aalen's additive regression

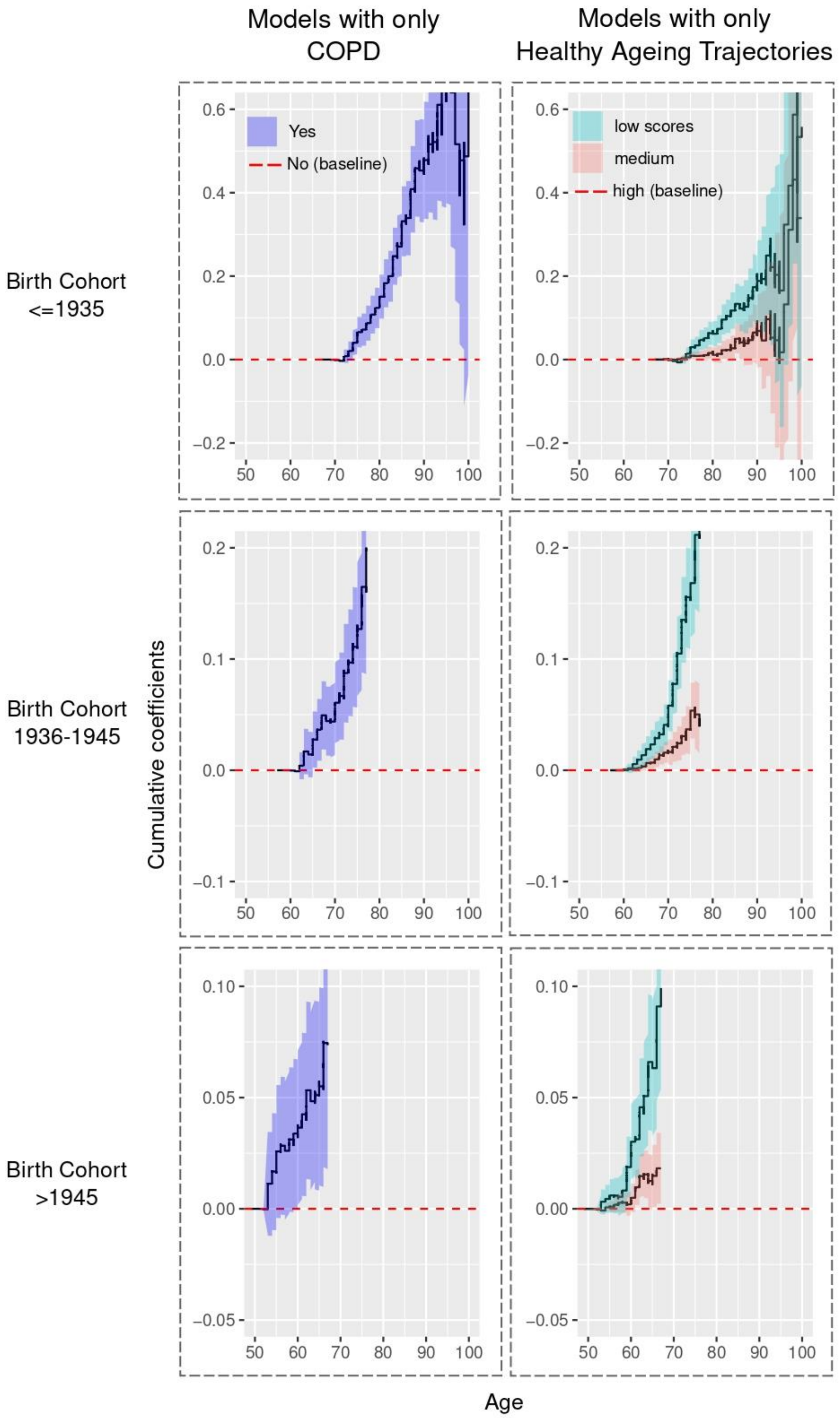

Supplementary Figure 3.

Figure 3.1. Estimates of cumulative excess risk of covariates from the Aalen's additive regression model by >1945 birth cohort sub-sample.

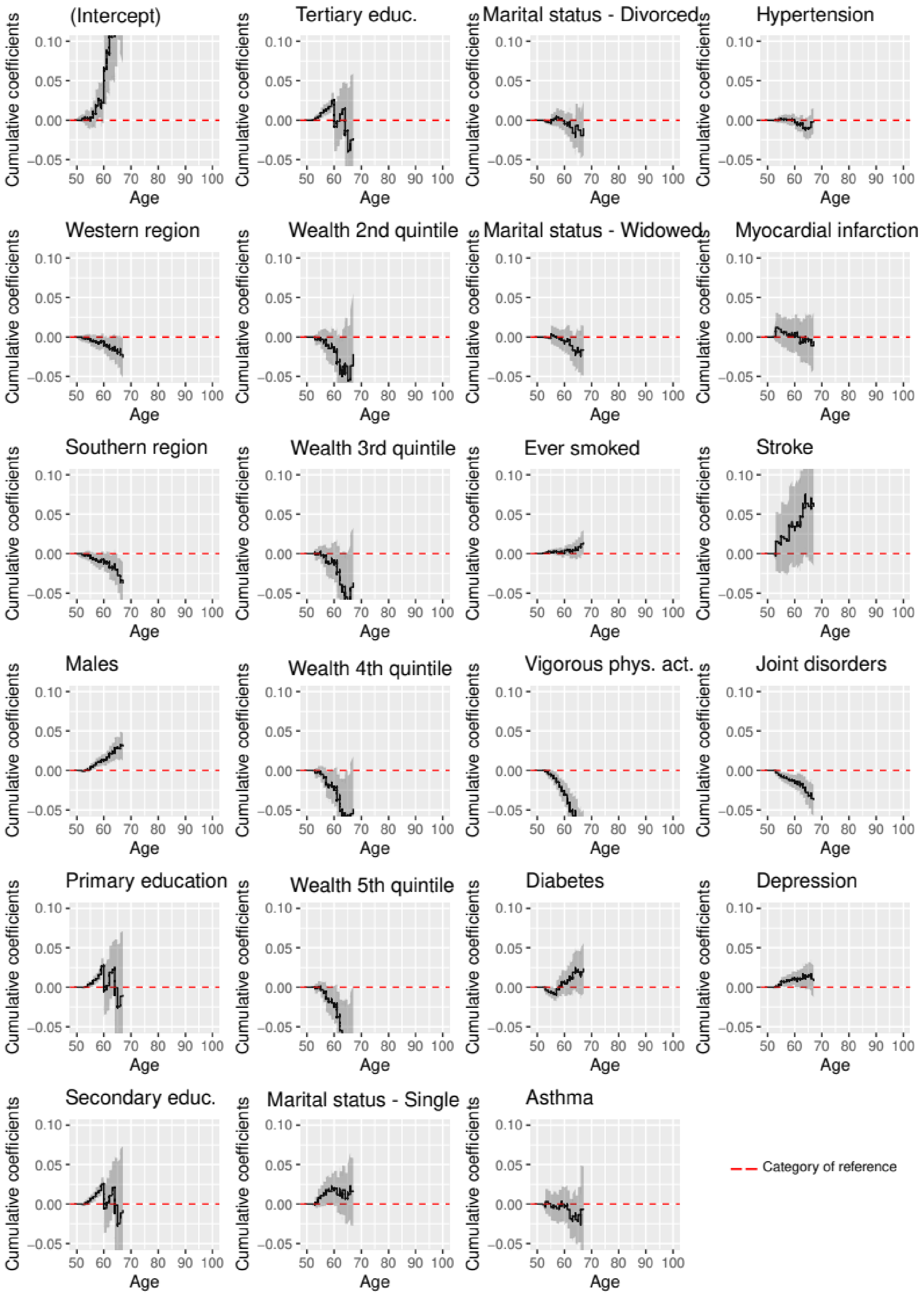

Figure 3.2. Estimates of cumulative excess risk of covariates from the Aalen's additive regression model by 1936-1945 birth cohort sub-sample.

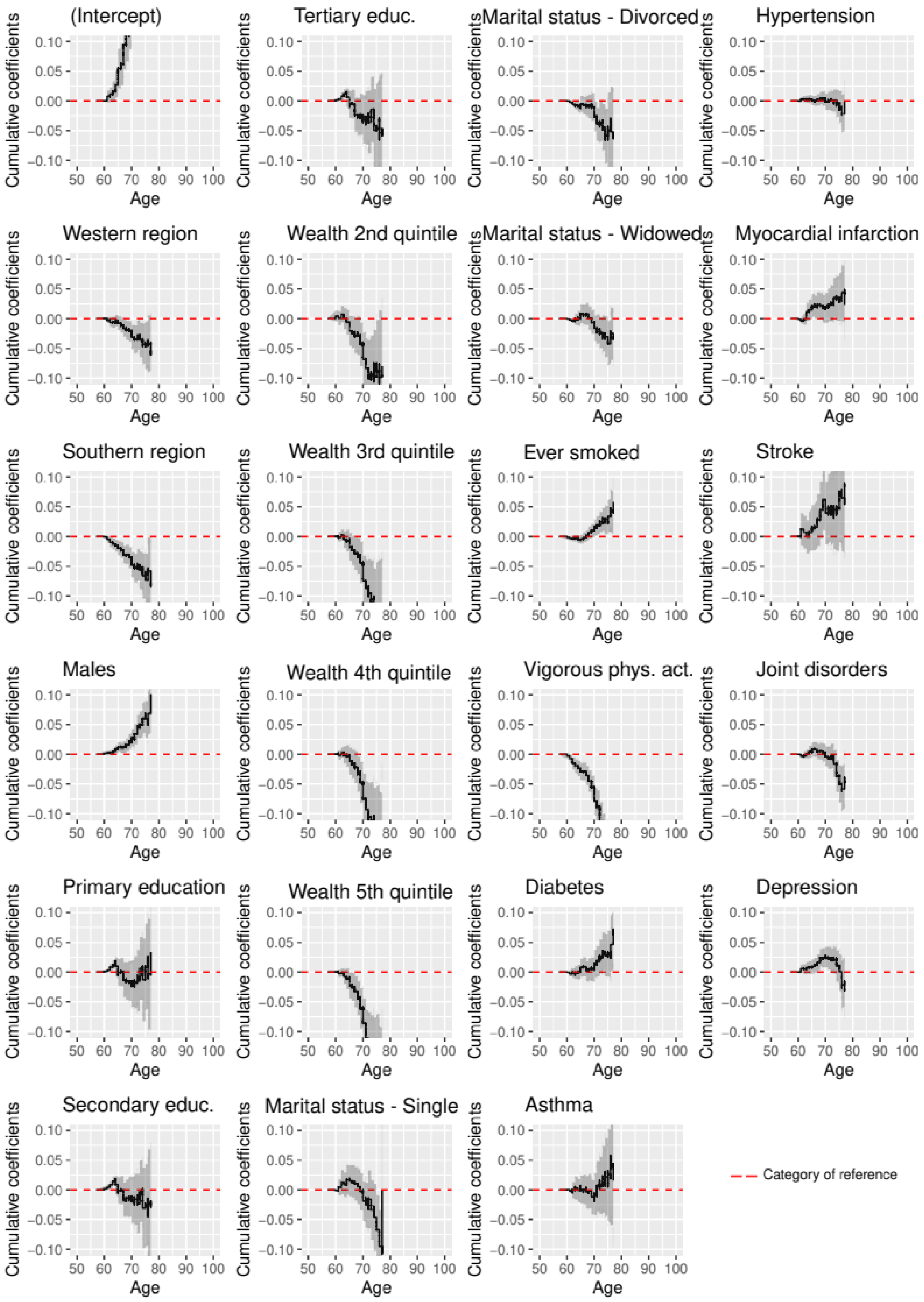

Figure 3.3. Estimates of cumulative excess risk of covariates from the Aalen's additive regression model by ≤1935 birth cohort sub-sample.

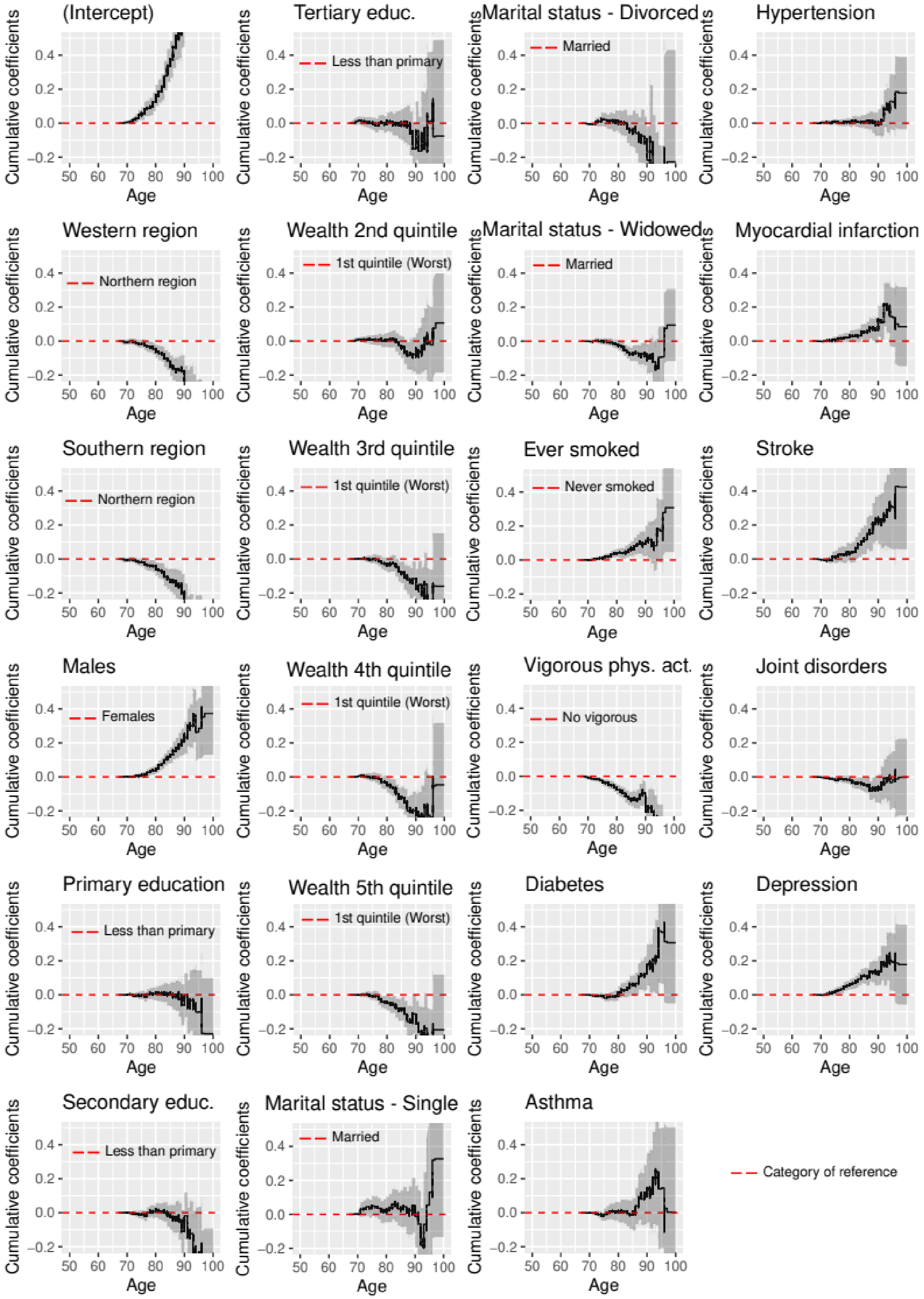

STROBE Statement—Checklist of items that should be included in reports of ***cohort studies***

	Item No	Recommendation	Page No
**Title and abstract**	1	(*a*) Indicate the study's design with a commonly used term in the title or the abstract<br>(*b*) Provide in the abstract an informative and balanced summary of what was done and what was found	1-2
**Introduction**			
Background/rationale	2	Explain the scientific background and rationale for the investigation being reported	3-6
Objectives	3	State specific objectives, including any prespecified hypotheses	5-6
**Methods**			
Study design	4	Present key elements of study design early in the paper	6
Setting	5	Describe the setting, locations, and relevant dates, including periods of recruitment, exposure, follow-up, and data collection	6
Participants	6	(*a*) Give the eligibility criteria, and the sources and methods of selection of participants. Describe methods of follow-up<br>(*b*) For matched studies, give matching criteria and number of exposed and unexposed	6-7
Variables	7	Clearly define all outcomes, exposures, predictors, potential confounders, and effect modifiers. Give diagnostic criteria, if applicable	7-9
Data sources/ measurement	8*	For each variable of interest, give sources of data and details of methods of assessment (measurement). Describe comparability of assessment methods if there is more than one group	7-9
Bias	9	Describe any efforts to address potential sources of bias	7-10
Study size	10	Explain how the study size was arrived at	6-7
Quantitative variables	11	Explain how quantitative variables were handled in the analyses. If applicable, describe which groupings were chosen and why	9-10
Statistical methods	12	(*a*) Describe all statistical methods, including those used to control for confounding<br>(*b*) Describe any methods used to examine subgroups and interactions<br>(*c*) Explain how missing data were addressed<br>(*d*) If applicable, explain how loss to follow-up was addressed<br>(*e*) Describe any sensitivity analyses	9-10
**Results**			
Participants	13*	(a) Report numbers of individuals at each stage of study—eg numbers potentially eligible, examined for eligibility, confirmed eligible, included in the study, completing follow-up, and analysed<br>(b) Give reasons for non-participation at each stage<br>(c) Consider use of a flow diagram	6-7
Descriptive data	14*	(a) Give characteristics of study participants (eg demographic, clinical, social) and information on exposures and potential confounders<br>(b) Indicate number of participants with missing data for each variable of interest<br>(c) Summarise follow-up time (eg, average and total amount)	10-11
Outcome data	15*	Report numbers of outcome events or summary measures over time	10-13

For peer review only

For peer review only - http://bmjopen.bmj.com/site/about/guidelines.xhtml

Main results	16	(*a*) Give unadjusted estimates and, if applicable, confounder-adjusted estimates and their precision (eg, 95% confidence interval). Make clear which confounders were adjusted for and why they were included<br>(*b*) Report category boundaries when continuous variables were categorized<br>(*c*) If relevant, consider translating estimates of relative risk into absolute risk for a meaningful time period	10-13
Other analyses	17	Report other analyses done—eg analyses of subgroups and interactions, and sensitivity analyses	Supplemental material
**Discussion**			
Key results	18	Summarise key results with reference to study objectives	17-18
Limitations	19	Discuss limitations of the study, taking into account sources of potential bias or imprecision. Discuss both direction and magnitude of any potential bias	16-17
Interpretation	20	Give a cautious overall interpretation of results considering objectives, limitations, multiplicity of analyses, results from similar studies, and other relevant evidence	13-18
Generalisability	21	Discuss the generalisability (external validity) of the study results	17-18
**Other information**			
Funding	22	Give the source of funding and the role of the funders for the present study and, if applicable, for the original study on which the present article is based	18

*Give information separately for exposed and unexposed groups.

Note: An Explanation and Elaboration article discusses each checklist item and gives methodological background and published examples of transparent reporting. The STROBE checklist is best used in conjunction with this article (freely available on the Web sites of PLoS Medicine at http://www.plosmedicine.org/, Annals of Internal Medicine at http://www.annals.org/, and Epidemiology at http://www.epidem.com/). Information on the STROBE Initiative is available at http://www.strobe-statement.org.

For peer review only
